# A Reconsideration of the Classification of the Spider Infraorder Mygalomorphae (Arachnida: Araneae) Based on Three Nuclear Genes and Morphology

**DOI:** 10.1371/journal.pone.0038753

**Published:** 2012-06-19

**Authors:** Jason E. Bond, Brent E. Hendrixson, Chris A. Hamilton, Marshal Hedin

**Affiliations:** 1 Department of Biological Sciences and Auburn University Museum of Natural History, Auburn University, Auburn, Alabama, United States of America; 2 Department of Biology, Millsaps College, Jackson, Mississippi, United States of America; 3 Department of Biology, San Diego State University, San Diego, California, United States of America; Field Museum of Natural History, United States of America

## Abstract

**Background:**

The infraorder Mygalomorphae (i.e., trapdoor spiders, tarantulas, funnel web spiders, etc.) is one of three main lineages of spiders. Comprising 15 families, 325 genera, and over 2,600 species, the group is a diverse assemblage that has retained a number of features considered primitive for spiders. Despite an evolutionary history dating back to the lower Triassic, the group has received comparatively little attention with respect to its phylogeny and higher classification. The few phylogenies published all share the common thread that a stable classification scheme for the group remains unresolved.

**Methods and Findings:**

We report here a reevaluation of mygalomorph phylogeny using the rRNA genes 18S and 28S, the nuclear protein-coding gene *EF-1γ*, and a morphological character matrix. Taxon sampling includes members of all 15 families representing 58 genera. The following results are supported in our phylogenetic analyses of the data: (1) the Atypoidea (i.e., antrodiaetids, atypids, and mecicobothriids) is a monophyletic group sister to all other mygalomorphs; and (2) the families Mecicobothriidae, Hexathelidae, Cyrtaucheniidae, Nemesiidae, Ctenizidae, and Dipluridae are *not* monophyletic. The Microstigmatidae is likely to be subsumed into Nemesiidae. Nearly half of all mygalomorph families require reevaluation of generic composition and placement. The polyphyletic family Cyrtaucheniidae is most problematic, representing no fewer than four unrelated lineages.

**Conclusions:**

Based on these analyses we propose the following nomenclatural changes: (1) the establishment of the family Euctenizidae (NEW RANK); (2) establishment of the subfamily Apomastinae within the Euctenizidae; and (3) the transfer of the cyrtaucheniid genus *Kiama* to Nemesiidae. Additional changes include relimitation of Domiothelina and Theraphosoidea, and the establishment of the Euctenizoidina clade (Idiopidae + Euctenizidae). In addition to these changes, we propose a “road map” for future sampling across the infraorder with the aim of solving many remaining questions that hinder mygalomorph systematics.

## Introduction

The infraorder Mygalomorphae, the trapdoor spiders, tarantulas, funnel web spiders and their kin, comprises 15 families that contain 325 genera and 2,675 nominal species [1]. The group is a diverse assemblage of relatively large, long-lived (15–30 years), ground dwelling spiders that build a diverse array of silk constructs used for prey capture, shelter, and protection [2]. Considered an ancient monophyletic group [3], [4], mygalomorphs retain several characteristics that are considered primitive for spiders, e.g., two pairs of book lungs, simple silk-spinning structures, etc. [5]. Many mygalomorph taxa are dispersal-limited [6], [7] and regionally-endemic, and have long been favorites of biogeographers [8]–[10]. Mygalomorph lineages have a deep evolutionary history as reflected by their relatively rich fossil record that extends back to the lower Triassic, with fossil representatives of several families dating to the mid-Cretaceous [11], [12]. Recent molecular clock analyses suggest that intra-familial divergences date to the Cretaceous [13], and inter-familial divergences may be as old as 300 Ma [14].

**Figure 1:**
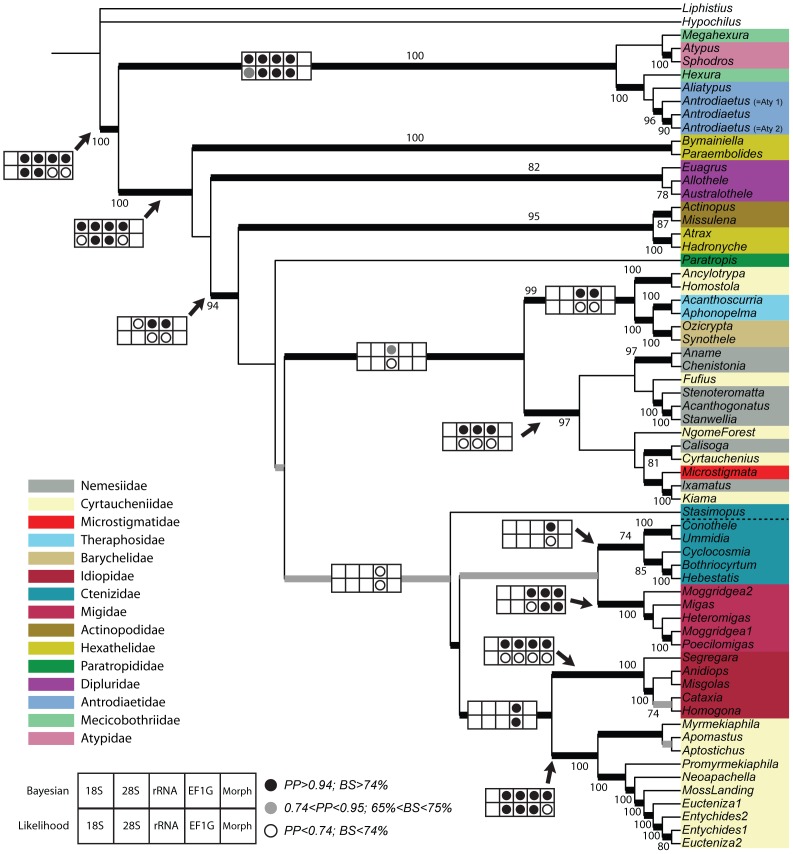
Summary of phylogenetic hypotheses based on molecular data partitions (28 S, 18 S, *EF-1γ*) using Bayesian inference. Dot plots indicate recovery and relative support for each node in separate analyses of the individual data partitions. For the combined gene analysis, thickened black and gray branches indicate posterior probability values that correspond to dot plot values in figure legend inset; values at nodes indicate bootstrap percentage values from the combined maximum likelihood RAxML analysis.

Over the past quarter century, mygalomorph systematics has received attention via four primary works that assess the monophyly and interrelationships of mygalomorph families (summarized in [5] Figure 1). Raven’s [15] work was seminal in that it was the first to apply an explicit cladistic framework (yet not computational) to evaluating relationships among mygalomorph families and genera using a set of defined morphological characters; this work remains the most comprehensive to date in terms of the breadth of taxa evaluated and serves as the fundamental framework for all future studies. Eskov and Zonshtein [16] followed shortly thereafter with an evaluation of some of Raven’s hypotheses and various critiques of the morphological characters he used to support his phylogeny and consequently proposed an alternative classification scheme. As has been the case for many years preceding [17]–[20], the composition of the Atypoidea and the placement of mecicobothriids was a major point of disagreement between the two classifications. Although their insights seem to have been largely ignored, Eskov and Zoshtein’s treatment was detailed and included a comprehensive discussion of persistent issues related to the efficacy of various mygalomorph characters, ambiguities related to how these characters have been scored, and how a number of the morphological characters used by Raven may be subject to strong selection as evidenced by associations with life history characteristics. These sentiments have been expressed by others [5], [21] and some of the very characters they discuss (e.g., carapace shape) have been shown to be ambiguously defined when evaluated quantitatively [22]. Three years later, Goloboff [23] produced the first cladistic analysis for the group using computational approaches to evaluate a set of 71 morphological characters scored for 42 taxa. His analysis was not only computational, but was also instrumental in highlighting the fact that a number of major problems in mygalomorph classification remain open for discussion; that is, the composition of the Atypoidea remained unresolved as did the monophyly of several families (e.g., Nemesidae, Dipluridae, and Cyrtuacheniidae). With the exception of recognizing a new clade (the Bipectina) and redefining Raven’s Crassitarsae, Goloboff conservatively left most major issues unresolved but supported the notion that atypoids excluded mecicobothriids.

Recognizing that morphological data alone appeared unable to definitively resolve relationships among the major mygalomorph groups and failed to achieve a consensus regarding family monophyly, Hedin and Bond [5] attempted the first molecular-based phylogenetic reconstruction for the infraorder. Their analysis, based on 18S and 28S ribosomal RNA genes, included representatives from 80 genera sampled across all 15 families (see Table S1). The phylogeny based on these data supported an atypoid clade that included Atypidae, Antrodiaetidae, and Mecicobothriidae but failed to support the monophyly of *most* families (e.g., Mecicobothriidae, Hexathelidae, Dipluridae, Cyrtaucheniidae, Ctenizidae, Nemesiidae, Microstigmatidae). Likewise, most of the proposed higher-level groupings were not supported (e.g., Fornicephalae, Domiothelina, etc.). As in other analyses [14], [21] the North American cyrtaucheniid subfamily Euctenizinae formed a monophyletic group. Following the lead of previous authors, Hedin and Bond chose to refrain from making nomenclatural changes as they felt that additional slowly evolving molecular phylogenetic markers, potentially combined with morphological data were needed to further resolve relationships among major taxa within this group.

In this paper, we present a reevaluation of mygalomorph relationships using a reduced sample of taxa based on our earlier rRNA data set coupled with an added single copy nuclear protein coding gene, *EF-1γ*, and the set of morphological characters used by Bond and Hedin [21]. The *EF-1γ* gene, developed by Ayoub et al. [14] shows promise for resolving mygalomorph relationships but to date has not been subjected to extensive sampling. The results reported herein are consistent with previous analyses based on fewer characters [5] or fewer taxa [14], [21] but show strong support for an atypoid clade that includes mecicobothriids, antrodiaetids, and atypids and strong support for the monophyly of the clade that includes all North American euctenizines. These results, while still wanting for increased taxonomic sampling, clearly indicate that considerable work remains to fully resolve mygalomorph classification – the monophyly of many mygalomorph families is called into question and the higher classification remains unresolved. Based on the phylogenetic hypothesis put forth here, we formally propose the elevation of the subfamily Euctenizinae to familial rank, transfer the Australian genus *Kiama* Main 1986 to Nemesiidae, and propose two higher-level clade designations to delineate newly identified groups. We conclude by discussing the status of each family and a few rather anomalous outcomes (e.g., the inclusion of microstigmatids within Nemesiidae) and we attempt to establish a framework for ultimately resolving the problems that plague mygalomorph classification.

## Materials and Methods

### Taxonomic Sampling and Data Preparation

Taxon sampling and data collection follows that described by Hedin and Bond [5]; specimens and GenBank accession data are documented in online supplemental material doi:10.1016/j.ympev.2006.05.017 [5] and Table S1 (this paper). As before, representatives of all mygalomorph families are included in the analysis. The taxon sampling scheme here differs from the previous based on rRNA gene data in that it is reduced from 99 to 62 ingroup taxa. We attempted to subsample the previous tree such that the major problems identified in the earlier rRNA analyses would remain germane in this study. The focus here was to reduce the number of taxa but increase the amount of data scored for each taxon. An additional outgroup taxon, (*Hypochilus*, sampled from the sister infraorder Araneomorphae) was included in the analysis along with a representative from the spider suborder Mesothelae (*Liphistius*). As before, two undescribed genera were included in the analysis (labeled as NgomeForest and MossLanding). Sampling was strengthened through the inclusion of additional euctenizine specimens (additional *Eucteniza* and *Entychides*) and the type genus for the family Cyrtaucheniidae (*Cyrtauchenius*).

Voucher specimens are preserved in 80% ethanol and tissues archived in RNAlater (Ambion Inc.) and stored at −80°C. Upon completion of our long term studies of mygalomorph phylogeny, specimens will be deposited in the collections of the National Museum of Natural History, Smithsonian Institution, Washington DC, the California Academy of Sciences, San Francisco, CA, and the Auburn University Museum of Natural History, Auburn, AL (AUMNH). Genomic DNAs were extracted and purified using Qiagen DNeasy Tissue Kit (Valencia, CA). Tissues and DNAs will be vouchered and archived in the AUMNH tissue collection. Procedures used to amplify and sequence the 18S and 28S rRNA genes are detailed in Bond and Hedin [21].

For *EF-1γ* approximately one-third of the sequences were obtained from GenBank [14] and the remaining data were generated using a two-step PCR amplification procedure following the protocols outlined in Ayoub et al. [14]. The first round of PCR reactions included a “touchdown” procedure using the primers EF1gF78 and EF1gR1258 under the following thermal cycler parameters: 18 cycles of denaturation at 94°C for 30 s, annealing at 58°C for 40 s (−1°C per cycle), and elongation at 72°C for 60 s; this was immediately followed by 16 cycles of denaturation at 94°C for 30 s, annealing at 42°C for 40 s, and elongation at 72°C for 60 s. The second round of PCR reactions made use of the product (1 µl) from the “touchdown” procedure and one of the following combinations of nested internal primers: EF1gF78/EF1gR856, EF1gF179/EF1gR1090, or EF1gF218/EF1gR1090; this consisted of 45 cycles of denaturation at 94°C for 30 s, annealing at 48°C for 40 s, and elongation at 72°C for 60 s. Both sets of PCR reactions included the following reagents (50 µl total reaction volume): 28.75 µl DNAase and RNAase free – deionized water (MP Biomedicals, Inc., Solon, OH), 5 µl dNTP mixture (2.5 mM each), 5 µl 10X Ex Taq buffer, 5 µl of each primer (2.5 µM), 0.25 *Taq* DNA polymerase (Takara Ex Taq, Fisher Scientific, Hampton, NH), and 1 µl genomic DNA (or 1 µl from product of first round of reactions). Unincorporated dNTPs, primers, and other impurities were removed from final PCR products using the High Pure PCR Product Purification Kit (Roche Diagnostics, Indianapolis, IN).

Amplification products were sequenced using an ABI Prism 377 or 3130 automated DNA sequencer (Applied Biosystems, Forest City, CA) using the ABI Big Dye Terminator version 3.1 Cycle Sequencing Ready Reaction Kit. Second-round PCR primers also served as sequencing primers [14]. All sequences were manually edited using the program Sequencher ver. 4.1.2 (Genecodes, Madison, WI).

Morphological characters scored are documented in Bond and Opell [24] and Bond and Hedin [21] and in Text S1.

### Multiple Sequence Alignment

*EF-1γ* sequence alignment followed the procedure outlined in Ayoub et al. [14]: sequences were translated and aligned using the default gap opening and gap extension costs in ClustalX ver. 2 [25]; the resulting protein alignment was used to facilitate alignment of individual nucleotides. As discussed in Hedin and Bond [5] alignment of the 28S data set was particularly problematic given high length variation among taxa. Rather than retain the alignments used in the previous published analysis (mainly as a consequence of the additional taxa) we chose to reevaluate the alignment of these data. Initial alignments were performed using the computer program MUSCLE version 3.6 [26] with the default gap opening and extension settings. The resulting alignment was then evaluated using the program Mesquite version 2.74 [27]; regions ambiguously aligned were further modified by delineating the block of problem sequences and then realigning using MUSCLE with the “Align Multiple Sequences” tool in Mesquite. Further minor adjustments were made manually as needed. Alignment of the 18S data set was far less problematic (considerably fewer indels). These data were likewise aligned using the computer program MUSCLE with only very minor manual adjustments in Mesquite to correct for obvious problems. Data sets were managed using Mesquite and are archived in the Dryad data repository.

### Phylogenetic Analyses

The program Kakusan4 [28] was used to determine the appropriate model of DNA substitution via the Bayesian information criterion (BIC) for phylogenetic analyses of each molecular partition (18S, 28S, and *EF-1γ*). The *EF-1γ* data were partitioned by codon position. A Mk+Γ model was used for phylogenetic analysis of the morphological data partition. We conducted six sets of analyses based on the following combinations of data sets: 18S, 28S, rRNA (18S +28s), *EF-1γ,* all genes (rRNA + *EF-1γ*), and total evidence (all genes + morphology). The complete concatenated data matrix comprised six partitions in total. Each data set was analyzed using Bayesian inference and maximum likelihood. Bayesian analyses were conducted using the computer program MrBayes ver. 3.1.2 [29], [30]. Tree searches comprised two independent runs of four Markov Chain Monte-Carlo (MCMC) chains run for 20–50 million generations, saving the current tree to file every 100 generations. Two independent simultaneous MCMC runs were performed to assess appropriate mixing of chains and to ensure topological convergence (split frequency ≤0.01). Convergence and stabilization of all parameters were visually inspected and verified in the program Tracer ver. 1.3 (available at http://evolve.zoo.ox.ac.uk/software.html?id  =  tracer). Topologies prior to –ln likelihood stabilization (as indicated by split frequency values and inspections in Tracer) were discarded as “burn-in” and clade posterior probabilities were computed from the remaining trees. The “total-evidence” (molecules and morphology) Bayesian topology presented herein represents the majority-rule consensus for all trees sampled in the posterior distribution.

Tree searches using maximum likelihood were conducted using RAxML ver. 7.2.8 [31], [32]. Partitioned RAxML analyses each comprised 1,000 random sequence addition replicates (RAS) using the commands “-q partition.txt”, “-# 1000” and “–m GTRGAMMA”. Analyses that included morphological partitions employed a Mk+Γ using the -m MULTIGAMMA and -K MK commands. Bootstrap support values were calculated using the same search parameters with 1,000 replicates. Bipartitions from the bootstrap analysis were then drawn on the best tree recovered from the RAS search.

### Nomenclatural Acts

The electronic version of this document does not represent a published work according to the International Code of Zoological Nomenclature (ICZN), and hence the nomenclatural acts contained in the electronic version are not available under that Code from the electronic edition. Therefore, a separate edition of this document was produced by a method that assures numerous identical and durable copies, and those copies were simultaneously obtainable (from the publication date noted on the first page of this article) for the purpose of providing a public and permanent scientific record, in accordance with Article 8.1 of the Code. The separate print-only edition is available on request from PLoS by sending a request to PLoS ONE, Public Library of Science, 1160 Battery Street, Suite 100, San Francisco, CA 94111, USA along with a check for $10 (to cover printing and postage) payable to "Public Library of Science".

In addition, this published work and the nomenclatural acts it contains have been registered in ZooBank, the proposed online registration system for the ICZN. The ZooBank LSIDs (Life Science Identifiers) can be resolved and the associated information viewed through any standard web browser by appending the LSID to the prefix "http://zoobank.org/". The LSID for this publication is: urn:lsid:zoobank.org:pub:69C4BCD9-FD19-4622-B500-B0358E4C6D5E.

## Results

### Data Characteristics

The aligned *EF-1γ* data set comprised 1386 aligned positions scored for 63 taxa (we were unable to obtain data for *Liphistius*). However, sequence length was variable due to primer fidelity inconsistencies across taxa; average proportion of missing data was 16%. The alignment was relatively straightforward with only a few regions containing gaps. The uncorrected base frequency composition appears to be homogenous (*Χ* ^2^ = 137.51, df = 186, P = 0.99; A = 0.32370, C = 0.18638, G = 0.22694, T = 0.26298). Pairwise distances (uncorrected p) across these data ranged from 0.012–0.345 with an average distance of 0.182. The aligned 18S rRNA data set comprised 1704 aligned positions scored for all 64 taxa. Sequences were relatively complete for most taxa except for *Hypochilus* (outgroup taxon), *Bymaniella*, and *Migas*; average proportion of missing data was 2%. Uncorrected base frequency composition appears to be homogenous (*Χ* ^2^ = 38.641238, df = 188, P = 1.00; A = 0.24749, C = 0.23135, G = 0.27619, T = 0.24497). Pairwise distances (uncorrected p) across these data ranged from 0.000–0.132 with an average distance of 0.019. The 28S rRNA data set comprised 2527 aligned positions scored for all 64 taxa. Sequences are relatively complete for all but *Hypochilus* and *Aliatypus* (each lacking the 3′ half of the region sequenced); average proportion of missing data was 5%. Uncorrected base frequency composition appears to be homogenous (*Χ* ^2^ = 193.685869, df = 189, P = 0.39; A = 0.20843, C = 0.26981, G = 0.33081, T = 0.19095). Pairwise distances (uncorrected p) across these data ranged from 0.001–0.390 with an average distance of 0.082. As discussed extensively by Hedin and Bond [5], alignment of rRNA genes was non-trivial. The 28S gene in particular contains a number of length-variable regions that appeared relatively ambiguous with respect to the initial MUSCLE alignment. Subsequently, these regions of perceived ambiguity (∼five based on visual examination) were realigned in Mesquite using MUSCLE (anchored on each end with unambiguously aligned regions) with marked improvement. As discussed below, phylogenetic results based on this alignment approach did not differ markedly from those we reported in earlier published works [5], [21] that relied on more extensive evaluations of alignment space.

Seventy-one morphological characters were scored for 63 taxa. *Hypochilus* was not evaluated due to the inapplicability of the majority of characters to non-mygalomorph taxa.

### Phylogenetic Analysis

The data and resulting tree files underpinning the analyses reported in this paper were deposited in NEXUS file format in the Dryad Data Repository at http://dx.doi.org/10.5061/dryad.7sq2j. Models of DNA substitution chosen for each of the data partitions, number of generations, burnin values and –ln log likelihood values for Bayesian and likelihood (RAxML) analyses are summarized in Table 1. With one exception the Bayesian runs converged quickly, however, additional generations were required to reach a standard deviation of split frequencies <0.01 for the 28S partition analysis. Figure 1 summarizes the phylogeny inferred from the molecular data partitions (Figures S1, S2, S3, S4, S5, S6). Congruence across each of the data partitions indicated by dot plots, shows that there was minimal agreement among the partitions, particularly at intermediate levels in the phylogeny. The 18S data set notably fails to recover all but a few of the nodes represented in the total evidence tree whereas the *EF-1γ* data set recovered many of them. Nodes supported in the rRNA combined data set are, not surprisingly, largely congruent with the results reported by Hedin and Bond [5]. All of the data partitions generally agree in their recovery of a monophyletic Atypoidea, Avicularioidea, and “euctenizine” clade (see Discussion below). As noted previously, rates of molecular evolution in the rRNA genes appear to be accelerated in a number of taxa (diplurids and Atypoidea, particularly *Megahexura* Fig. 1); these unusually long branches are not observed in the *EF-1γ* gene trees. Figure 1 also shows that the concatenated molecular data analysis is largely incongruent with the likelihood and Bayesian analyses of the morphological data. The morphology partition only recovers the molecular delineated clades Avicularioidea, “Euctenizinae”, Idiopidae, and Migidae. The morphological analysis does recover a monophyletic Ctenizidae and places *Cyrtauchenius* among the Domiothelina taxa (Figure S7); clades not recovered in any of the molecular analyses.

**Table 1:** Summary of phylogenetic analysis models, run parameters, and likelihood values [arithmetic (upper) and harmonic means (lower) for Bayesian runs] for each data partition, and combined analyses.

Data Set	Substitution Model(s)	Ngens burnin	-ln likelihood value (Bayes)	-ln likelihood value (RAxML)
**18S**	SYM+Γ	20×10^6^	−6750.89	6686.575467
		5×10^6^	−6901.19	
**28S**	SYM+Γ	50×10^6^	−20910.88	−20848.731740
		40×10^6^	−20969.34	
**rRNA**	SYM+Γ (all)	20×10^6^	−28314.69	−27967.730323
		5×10^6^	−28367.89	
**EF1G**	K80+Γ (1)	20×10^6^	−16462.53	−16435.386678
	GTR+Γ (2,3)	12×10^6^	−16512.63	
**All genes**	–	20×10^6^6×10^6^	−45961.34−46017.60	−45435.580124
**Genes+morph**	–	30×10^6^	−48415.78	−48801.099709
	Mk+Γ	5×10^6^	−48471.71	

**Figure 2:**
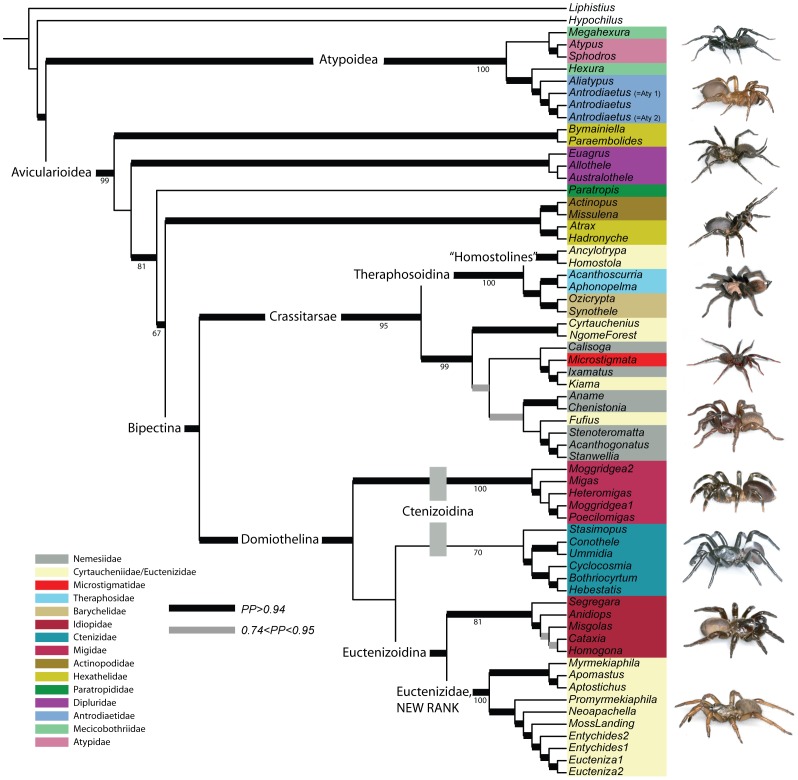
Total evidence phylogenetic hypothesis and revised classification based on Bayesian inference analysis. Thickened black and gray branches indicate posterior probability support values; values at nodes indicate bootstrap percentage values from the combined maximum likelihood analysis conducted in RAxML. Pictured taxa from top of figure to bottom: male *Sphodros atlanticus* (Atypidae); *Antrodiaetus unicolor* (Antrodiaetidae); *Namirea planipes* (Dipluridae); *Atrax robustus* (Hexathelidae); *Aphonopelma* sp. (Theraphosidae); male *Microstigmata longipes* (Microstigmatidae); male *Kiama lachrymoides* (Cyrtaucheniidae – transferred to Nemesiidae); *Moggridgea* sp. (Migidae); male *Ummidia* sp. (Ctenizidae); *Aptostichus* sp. (Cyrtaucheniidae – removed to Euctenizidae).

Figure 2 summarizes the total evidence tree topology (all genes and morphology). The total evidence tree is in general agreement with the molecular tree but notably recovers a monophyletic Ctenizidae. The molecular analyses fail to unite the South African genus *Stasimopus* with other ctenizids, a clade recovered only in the analysis of the morphological partition (noted above).

The results are not particularly sensitive to analytical approach (Bayesian vs. likelihood; see Figures S1, S2, S3, S4, S5, S6, S7). The respective analyses of the 18S data partition both resulted in an Atypoidea clade that was sister to the remaining taxa. Both analyses recovered few clades with any notable support. The 28S data partition, taken alone, faired slightly better in its recovery of a number of major family level groups. A euctenizine and Nemesiidae clade (including *Kiama* and *Microstigmata*) were recovered in the Bayesian and likelihood analyses, although the latter analysis only weakly supported the nemesiids. The combined likelihood and Bayesian analysis of the rRNA genes and *EF-1γ* were largely congruent. The most notable exception was the relationships among the more “basal” mygalomorph taxa in the *EF-1γ* gene trees; that is, the relative positions of hexathelids, diplurids, *Paratropis*, and actinopodids varied among the two approaches and were generally weakly supported. Likewise, the Bayesian and likelihood analysis of the concatenated molecular data sets were largely congruent. The only notable exceptions were the position of *Paratropis* relative to Hexathelidae + Actinopodidae and Dipluridae and the generally higher branch support in the Bayesian analysis. The total evidence (all genes and morphology combined) likelihood and Bayesian trees were largely congruent with two notable exceptions. First, the likelihood analysis failed to recover the hexathelid + actinopodid clade observed for many of the other partitions. Indeed, the likelihood analysis placed actinopodids as sister to migids, a phylogenetic position more consistent with past hypotheses [15], [23]. Second, the likelihood analysis also united diplurids and some hexathelids (*Bymainiella* and *Paraembolides*) as sister groups, whereas the Bayesian analysis retained these as a grade of lineages sister to remaining mygalomorph taxa.

## Discussion

### Preferred Phylogenetic Hypothesis

Our preferred phylogenetic hypothesis, based on all of the evidence available to us at this time (molecules and morphology) is summarized as the Bayesian inference tree in Figure 2. As noted earlier, this hypothesis is largely congruent with the RAxML likelihood tree but differs mainly in the placement of the two actinopodid taxa. The combined genetic data clearly support (Fig. 1) the placement of these taxa as sister to the “hexathelid” genera *Atrax* and *Hadronyche*; thus, the discrepancy here is likely related to the apparently overwhelming strong signal contributed by the morphological data in the RAxML analysis. It is worth noting that the conflict in these data appear to substantially impact support values further up the tree (i.e., most of the intermediate level nodes in the Domiothelina clade are weakly supported).

Our hypothesis put forth here is consistent with previous published results and indicates that a stable systematic framework for the Mygalomorphae has still not yet been achieved. As we discuss in detail below, almost half of the 15 families are either para- or polyphyletic and thus major changes to mygalomorph classification will likely be warranted in the future. Unfortunately, sampling to date precludes (conservatively) making major changes at this time; however, these results clearly define focal points for future collecting and data sampling efforts. As has been commented on by a number of authors [16], [22]–[24], certain morphological characters shared among ancient mygalomorph lineages likely reflect shared ecological characteristics rather than phylogenetic history. It is our opinion that future efforts must focus on expanded taxonomic and gene sampling rather than harvesting additional morphological characters. In the systematics section below, we outline what nomenclatural changes we believe are warranted at this time given our sampling scheme, apparent strength of the hypothesis, and consistencies with previous analyses.

### Mygalomorphae Systematics

#### Atypoidea

The Atypoidea, the clade that includes the Atypidae, Antrodiaetidae, and Mecicobothriidae, is strongly supported by all of the various analyses of each gene partition (Figs. 1 and 2). As noted by Hedin and Bond [5], this hypothesis is historically one of the most controversial in mygalomorph systematics. While their data supported the Atypoidea, they also suggested that the molecular data was in conflict with the morphological data and that additional DNA evidence would be required to corroborate the hypothesis; that is, long branch attraction may have played a role in uniting these taxa in the rRNA gene trees. Given the consistencies with previous data, the additional *EF-1γ* data, and relatively strong bootstrap and Bayesian posterior probability support, the status of the Atypoidea as a monophyletic group that is the sister group to all other mygalomorphs seems secure.

#### Antrodiaetidae

Antrodiaetid monophyly is well supported by our results (Fig. 2) and is generally not a point of contention. The family is Holarctic in distribution and currently comprises two genera composed of 33 species [1], [33]; it has received considerable taxonomic and phylogenetic treatment in recent years [13], [33]–[38].

#### Atypidae

The family Atypidae currently comprises three genera composed of 48 nominal species [1]. Our phylogenetic hypothesis supports the monophyly of the family but the sampling has a number of significant shortcomings. First, it includes only two genera, omitting any samples from the genus *Calommata*, an oversight in all recent studies of mygalomorph higher classification [39]. Based on palpal affinities, Gertsch and Platnick [40] considered *Calommata* likely sister to *Sphodros*, however, they noted that it is perhaps one of the “world’s most bizarre spider genera”. Not surprisingly the placement of the genus in Atypidae has been questioned [41]. Second, our sampling of *Atypus* is sparse, comprising the single North American representative (*Atypus snetsingeri* Sarno) of this widely distributed Holarctic genus. Consequently, the family requires more sampling that at a minimum would include *Calommata* and additional *Atypus* species before we could be confident that the family is monophyletic.

#### Mecicobothriidae

Mecicobothriidae is a relatively small family containing nine species placed among four genera; one genus, *Megahexura*, is monotypic [1]. As discussed above, placement of mecicobothriids in the Atypoidea has until now been contentious, however, monophyly of the family still appears unresolved (Fig. 2). Our present analysis includes representatives of two genera, *Hexura* and *Megahexura* thus omitting *Hexurella* and *Mecicobothrium*. The inferred phylogeny places *Megahexura* as sister to atypids and *Hexura* as sister to antrodiaetids. These results are generally consistent with the independent analyses of the rRNA and *EF-1γ* data partitions; however, they are not necessarily surprising given the recognized morphological affinities among the various mecicobothriid genera, antrodiaetids, and atypids [18]. That said, as noted earlier there may be issues with respect to long branch attraction, particularly for *Megahexura* (rRNA but not *EF-1γ*). Without question, definitively resolving the monophyly (or lack thereof) of Mecicobothriidae requires inclusion of *Mecicobothrium* (preferably *M. thorelli*, the type species for the genus) and *Hexurella*.

#### Avicularioidea – Bipectina – Crassitarsae

With the exception of some relimitation, we generally retain the existing higher-level clade structure for the infraorder. As discussed by Goloboff [23], the family name Aviculariidae was used by Simon [17] as the designation for all mygalomorphs except atypids, antrodiaetids, and mecicobothriids but was later reconfigured to include the mecicobothriids [42]. Consequently, we retain the older delimitation of Avicularioidea in favor of the Orthopalpae (Fig. 2). We follow Hedin and Bond [5] in recognizing Goloboff’s [23] Bipectina, relimited here to exclude the family Paratropididae; our sampling does not allow us to make any determinations with respect to the inclusion of diplurines (Dipluridae) in the clade. Like Goloboff [23], we retain the Crassitarsae but relimit it to include the Microstigmatidae (likely to be included in Nemesiidae, see below) and exclude paratropidids. It is probably not worthwhile to discuss here in detail what taxon sampling would most likely resolve these issues, however, it suffices to say that increased sampling to address most questions about family monophyly will contribute significantly towards resolving higher-level issues across the Mygalomorphae.

#### Hexathelidae – Dipluridae

Hexathelids and diplurids collectively form a grade of taxa sister to the Bipectina clade and require considerable work if a stable classification scheme is to be achieved. Hexathelidae is a relatively diverse group comprising 12 genera composed of 105 species [1]. Problems within the family were discussed extensively by Hedin and Bond [5]; the previous results indicating Hexathelidae polyphyly are supported here (Fig. 2). Although we remain confident that macrotheline and hexatheline taxa do not form a single clade, additional sampling would be required before formal changes would be warranted. However, based on these data it is likely that the family will be divided, at the very least, into two separate groups. Our previous analyses [5] based on rRNA gene data included more extensive sampling (*Porrhothele* and *Macrothele*) than this study, but were relatively inconclusive with respect to how the group may ultimately be divided because *Porrhothele* and *Macrothele* (macrothelines) did not share a common ancestor. Target taxa for future sampling must include *Hexathele, Teranoides, Macrothele*, *Mediothele,* and *Porrhothele* species.

The Dipluridae, likewise problematic, is a relatively diverse family comprising 25 genera with 179 species [1]. Somewhat surprisingly the diplurid taxa form a single clade in the analysis reported herein (Fig. 2) whereas the group formed a paraphyletic grade of taxa in the previous study [5]. While the sampling scheme of Hedin and Bond [5] included a number of additional genera, our sampling here represents the breadth of diversity from that study. Unfortunately, both studies include only euagrine taxa and as a result, few conclusions can really be drawn regarding diplurid monophyly. Future work must include at a minimum *Diplura, Microhexura,* and masteriine and ischnotheline representatives.

#### Paratropididae

The family Paratropididae is a small family comprising only four genera composed of eight species [1]. The group is rather enigmatic and appears to be difficult to place phylogenetically [5], [15], [23]. As noted previously [5], our sampling scheme that includes only a single generic representative is obviously inadequate to evaluate the monophyly of the family. However, these data (Fig. 2) are in conflict with morphological based hypotheses [15], [23] placing the family as sister to theraphosids and barychelids. Additional sampling of the remaining genera (minimally *Anisaspis* and *Melloina*) will likely clarify the phylogenetic placement of this family, but we have serious doubts that the Theraphosoidina or Theraphosoidea clades will be supported as currently defined.

#### Cyrtaucheniidae

Cyrtaucheniidae in its present form *must* be abandoned in favor of a considerably relimited construct. This family represents one of the largest and most difficult problems for resolving mygalomorph classification. All analyses since Raven [15], both morphological [23], [24] and molecular [5], [21], unequivocally indicate that the family is polyphyletic. As currently configured, it comprises 18 genera composed of 134 species [1] and includes a considerable degree of morphological diversity (Bond persn. obs.). Based on our sampling of 12 of the 18 described genera (plus two more putatively undescribed genera) and preferred phylogenetic hypothesis (Fig. 2), the family minimally encompasses taxa representing four unrelated lineages: (1) the lineage composing South African genera that are sister to theraphosids and barychelids (labeled informally here as “Homostolines”); (2) cyrtaucheniids (*sensu stricto*); (3) at least two lineages embedded within nemesiids (*sensu lato*); and (4) a clade that includes all North American euctenizines. However, it is worth noting that *Cyrtauchenius* and the undescribed NgomeForest genus from South Africa do not form a single clade in any of the molecular analyses (Fig. 1) and thus are more likely to be ultimately allocated to Nemesiidae.

A conservative approach to resolving the nomenclatural difficulties that the family Cyrtaucheniidae presents is going to be non-trivial and will require collecting additional, difficult to obtain taxa (e.g., *Rhytidicolus* and *Acontius*). While a number of issues appear easy to resolve – e.g., status of Euctenizinae and placement of *Kiama* – any nomenclatural emendations such as these essentially leave remaining a taxonomic construct further rendered artificial that simply serves as a placeholder (i.e., dumping ground) for taxa that are essentially *incertae sedis*. Alternatively, a more liberal and imperfect (in terms of taxonomic sampling) approach could be taken – a new family could be erected to accommodate *Ancylotrypa* and *Homostola* and the remaining genera could be collapsed into Nemesiidae. The latter aspect of this approach, while somewhat bold, would not be in conflict with either our preferred hypothesis of relationships (Fig. 2) or the analyses based on molecular data alone (Fig. 1).

While we are confident that Cyrtaucheniidae must ultimately be disassembled, for a number of reasons we are unsure that it would be in the best interests of nomenclatural stability to dissolve the family entirely at this time. The current taxonomy and systematics of the South African genera *Homostola* and *Ancylotrypa* does not make us confident that erecting a new family on the basis of samples from these two species would be particularly prudent. That is, we are not at all sure that either genus is monophyletic and/or that the specimens from which we sampled were of species that accurately and precisely represent the limits of these genera. We likewise think that the same logic applies for *Fufius* and *Cyrtauchenius*; the “cyrtaucheniid” species from Ngome will be placed into Nemesiidae when it is formally described. Given these ambiguities, it is likely that such changes, if made now, would still remain one step away from being optimal; more extensive future studies would require another round of emendation. As such we would likely do little to advance mygalomorph systematics through the wholesale dismantling of the family based on these data.

As mentioned above, two significant nomenclatural changes are unequivocally supported by these data. First, we formally propose the transfer of the monotypic Australian genus *Kiama* to the family Nemesiidae (Appendix A). *Kiama* shares a number of features to include a unique pustulose cuticle and similarities in fine spinning structures with *Microstigmata* and *Ixamatus* (see Figure 3 in [24] and Fig. 2). Second, we propose the elevation of Euctenizinae to the family rank (see below). Placement of the remaining cyrtaucheniid taxa will require extensive sampling of the species- and generic-level diversity within the Cyrtaucheniinae and Aporoptychinae.

#### Euctenizidae NEW RANK

The newly established family Euctenizidae comprises seven genera composed of 32 species (Appendix B). As discussed by Bond and Hedin [21], morphological synapomorphies that are not homoplasious when considered across all mygalmorphs are lacking. As such, the earlier study proposed a combination of characters that supported “euctenizid” monophyly – a wide and deep foveal groove, asymmetrical female tarsal scopulae, unique arrangement of silk spigots on the tip of the posterior lateral spinnerets, two unique silk spigot types on the posterior median spinnerets, the presence of preening combs on metatarsus IV, femur IV with a distinctive patch of dense spines, male palpal femur with a distinct dorsal spine row, and spermathecae with a basal lateral extension (not multi-lobed). Despite rather tenuous morphological support, a single Euctenizidae clade is supported in every morphological, molecular, and combined analysis; evidence for this clade appears *unequivocal*. The group is restricted in distribution to North America and includes undescribed taxonomic diversity (additional species and genera). Comprising two strongly supported subgroups, intergeneric relationships appear relatively stable within the family. The Apomastinae (NEW SUBFAMILY) comprises *Apomastus, Myrmekiaphila*, and *Aptostichus*; Euctenizinae is relimited here to include all remaining euctenizid genera.

#### Crassitarsae – Theraphosoidina

Defined by Raven [15], the Crassitarsae clade comprises nemesiids, paratropidids, barychelids, and theraphosids. As already discussed, our analyses (Figs. 1 and 2) do not support the sister group relationship of paratropidids and the Theraphosoidina. Consequently, we relimit the Crassitarsae (Fig. 2) to exclude paratropidids. The Theraphosoidina likewise is relimited to exclude paratropodids but will likely include a third family that comprises “Homostolines” (pending future enhanced sampling); Theraphosoidea (theraphosids + paratropoidids) is no longer a recognized grouping.

#### Theraphosidae – Barychelidae

The families Barychelidae and Theraphosidae (Fig. 2) comprise the highest nominal diversity among mygalomorphs –44 genera with 303 species and 120 genera with 937 species, respectively [1]. Although both families appear to be monophyletic, these results are based on severely limited sampling – two theraphosid species representing one subfamily (the New World Theraphosinae) and two Australian barychelid genera. The two families are recognized from other mygalomorphs by having distinct claw tufts and very well developed leg scopulae [15]. The somewhat weak characters that distinguish theraphosids are described as the presence of a prominent anterior lobe on the maxilla and an increased density of labial and maxillary cuspules; barychelids are considered to have a weak anterior lobe and generally fewer cuspules. Moreover, barychelids differ slightly in their spinneret morphology by having a domed posterior lateral distal segment. Given the general weakness in these characters, some of which are shared widely across mygalomorphs, Raven [15] recognized that there really were not any well-supported synapomorphies for Theraphosidae (*sans* Barychelidae). The lack of character support can be attributed primarily to problems related to the position of the subfamily Ischnocolinae (the sister group to all other theraphosids), contributing heavily to a troubled taxonomic history. Several ischnocoline genera have shifted between the two families [15], [43] suggesting that theraphosids and barychelids may not be so distinct.

Despite the fact that the monophyly and placement of the Theraphosoidea within mygalomorphs will likely not change, an enhanced sampling of genera from both groups with a particular emphasis on the Ischnocolinae may resolve the status of both families. That is, do theraphosids and barychelids represent one or two distinct clades? Candidate barychelid genera include the following: the Australasian genus *Monodontium*, thought to represent the sister genus to all other barychelids [44]; the African genus *Brachionopus* – formally Theraphosidae [43]; the Indian genus *Diplothele*; the African genus *Pisenor*; and the New World genera *Psalistops* and *Reichlingia* (originally described as a theraphosid [45]). In addition to taxa from other subfamilies, as already mentioned, future sampling from across the Theraphosidae must focus on the ischnocoline genera, including: the New World genus *Holothele* (transferred to Theraphosidae from Dipluridae by Raven [46]; the African *Heterothele*; and the North African/Middle Eastern *Chaetopelma*.

#### Nemesiidae – Microstigmatidae

The family Nemesiidae comprises 42 genera composed of 355 species. The far less diverse and somewhat enigmatic family Microstigmatidae contains only seven genera and about 15 species. Our results are consistent with the conclusions of Goloboff [23] that the family Nemesiidae is likely paraphyletic. Although Goloboff indicated that the family may need to be split at some point in the future, our results are more consistent with his alternative suggestion that the microstigmatids and close relatives (e.g., *Kiama, Angka, Ixamatus*) be placed into a new subfamily within the Nemesiidae. To fully resolve the limits of the Nemesiidae + Microstigmatidae clade, future sampling needs to focus on additional microstigmatid genera but must also include a number of representatives from other nemesiid subfamilies (e.g., Bemmerinae and Diplothelopsinae).

#### Domiothelina – Euctenizoidina – Migidae – Idiopidae – Ctenizidae

The Domiothelina clade as defined by Raven [1] includes four families – Migidae, Actinopodidae, Ctenizidae, and Idiopidae. Raven considered actinopodids and migids sister groups that composed the clade Migoidea; the Ctenizoidina comprised the Migoidea plus Ctenizidae. Our analysis (Figs. 1 and 2) does not support any of these groupings (as defined by Raven). We thus propose retaining Domiothelina to include euctenizids and tentatively exclude actinopodids (see below). The Ctenizoidina is tentatively retained here (excluding actinopodids) because it is supported in the molecular analyses (Fig. 1, but see discussion below regarding ctenizid monophyly with respect to *Stasimopus*). We formally recognize here the unique group of Euctenizidae + Idiopidae as the Euctenizoidina clade (also in the molecular analysis, (Fig. 1). This new subgroup likely reflects the previously unrecognized morphological affinities shared by some euctenizid and idiopid taxa (e.g., modifications of the male palpal tibia and relatively dense scopulae). The Migoidea will likely be abandoned given the apparently distant phylogenetic placement of actinopodids. Despite these changes, the robustness of the Domiothelina clade is questionable given that it is only weakly supported in the Bayesian analyses of the molecular data alone; that is, the group is only supported in the analyses that consider morphology. Taxon sampling is relatively dense across this group and it is therefore our opinion that any improvements are likely to be gained through additional characters (genes) rather than through enhanced sampling of taxa.

The family Migidae comprises 10 genera composed of 91 species [1], [9]. As discussed by Hedin and Bond [5], this family is generally distinctive both morphologically and behaviorally but was not supported strongly by the rRNA data. The results shown here (Figs. 1 and 2) indicate relatively strong support for the family in the *EF-1γ* and combined analyses, but as noted in the earlier study, a monophyletic *Moggridgea* is never recovered.

The family Ctenizidae, the group typically thought of as “trapdoor spiders”, comprises 125 species distributed among nine genera [1] Species-level diversity in this group will likely double as genera such as *Ummidia* are revised. With the exception of the rather difficult to place South African genus *Stasimopus*, the family appears to form a generally well-supported clade (although sampling is rather limited). However, it is worth noting that a monophyletic Ctenizidae (save *Stasimopus*) is recovered only in the *EF-1γ* and combined analyses. As was the case for migids, the *EF-1γ* data set provides the additional signal necessary to recover this group. However, only the total evidence phylogeny (Fig. 2) recovers Ctenizidae with *Stasimopus* – a seemingly difficult genus to place as evidenced by it affinities with migids [5]. As noted by Raven [15], *Stasimopus* shares a number of features with other non-ctenizid taxa, particularly migids (e.g., characteristically wide ocular area). As such it is not surprising that the genus presents some difficulties for the ctenizids, migids, and the overall structure of Domiothelina phylogeny.

Raven [15] designated two subfamilies within Ctenizidae – Pachylomerinae and Ctenizinae. Our sampling includes taxa from both (*Bothriocyrtum, Cyclocosmia,* and *Stasimopus* from the former; *Ummidia, Conothele,* and *Hebestatis* the latter). The current hypothesis (Fig. 2) of relationships does not support a monophyletic ctenizine clade with the inclusion of *Hebestatis*. Representatives of the remaining genera *Cteniza, Cyrtocarenum,* and *Latouchia* would likely contribute significantly to resolving the status of Ctenizidae and its intergeneric relationships.

Finally, the Idiopidae is a rather diverse mygalomorph family that currently comprises 22 genera composed of 302 species [1]. The monophyly of the family is well supported by most of the data (Fig. 2). Contrary to Hedin and Bond [5], association with other domiotheline taxa has reasonably solid support here. Although we doubt that additional sampling will bring idiopid monophyly into question, it is worth noting that the group’s diversity is not entirely represented in our study (e.g., genysine taxa are entirely absent). Consequently, future efforts should include a number of additional genera (e.g., *Idiops, Neocteniza,* and *Genysa*) that would provide a more rigorous test of familial monophyly but would also further resolve generic relationships and subfamily delimitation.

#### Actinopodidae

These results presented here (Figs. 1 and 2) raise some interesting questions with regards to the status and placement of actinopodids. The family comprises only three genera composed of 40 species [1]. Although not recovered in all analyses (e.g., total evidence likelihood analyses, see above), our preferred hypothesis (Fig. 2) places actinopodids as the sister group to the hexathelid taxa rather than within the Domiothelina. Given the morphological affinities of actinopodids with other domiotheline taxa, their placement elsewhere in the phylogeny seems rather suspect. Nevertheless this unexpected pairing is strongly supported in the *EF-1γ* and combined analysis; because the relationship is recovered for two independent genera and samples, we do not suspect contamination or some other technical problem to be an issue. Moreover, *Missulena* venom proteins are conspicuously similar to those of *Atrax* [47], further suggesting that these taxa may be more closely related than previously thought. Taxon sampling across actinopodids is not particularly dense but does include two of the three described genera. Given just how anomalous these results are we feel that it would be worthwhile to sample additional species before lending full support to such an altered view of mygalomorph phylogeny.

### Conclusions

The infraorder Mygalomorphae represents an interesting and diverse group that have retained a number of features generally considered primitive for the order Araneae. However, mygalomorphs have received little attention with respect to evaluation of family limits and higher classification when compared to the many higher level studies in the Araneomorphae. The results of the study we report herein demonstrate that a considerable amount of work remains before the internal structure across the infraorder is resolved and family monophyly is achieved. As discussed earlier, the family Cyrtaucheniidae represents one of the greatest obstacles to solving many of the major points of higher classification; the family unequivocally has served as a prototypical dumping ground for difficult to place genera and other taxa. Although our sampling was inadequate to reconcile the placement of all cyrtaucheniid taxa, we are confident in the establishment of the Euctenizidae as a newly recognized spider family. The monophyly of the clade has been well established in a number of past studies and is strongly supported here. In addition to these problems, of the remaining 14 families, more than half of these are problematic with respect to phylogenetic placement and/or monophyly. Consequently, the amount of work that lies ahead is considerable and will require a carefully designed sampling scheme. Moreover, it seems clear that while a molecular approach to evaluating mygalomorph phylogeny is justified, we still have not managed to achieve a high level of precision from the genes we are employing. The recent advances in next generation sequencing technologies may rapidly abrogate the bottleneck that non-model organisms like these present for issues related to gene discovery. Such future studies will need to balance the need for new genes with the need to be more inclusive with respect to taxon sampling such that some level of confidence can be achieved when making major nomenclatural changes (e.g., restructuring families).

As with any analysis of this type, ours suffers from a number of shortcomings. As already demonstrated and discussed, we still lack a sufficient amount of data to be reasonably confident in all of the recovered relationships. Our general approach has been to remain relatively conservative in making major nomenclatural emendations in the interests of minimizing the total number of changes over time. That is, while we may be confident that a problem exists (e.g., the polyphyly of the Cyrtaucheniidae), we do not always have complete confidence in what changes will likely withstand the test of additional data. With respect to the actual characters, it seems clear that we have not yet obtained a sufficient number of genes with signal at the appropriate phylogenetic levels to fully resolve, with reasonable support, all of the mygalomorph branches. Based on this study and others, standard genes used in arachnid systematics (rRNA genes, mitochondrial DNA) are not going to provide the necessary signal – clearly, a more comprehensive genomics-based approach is likely necessary. Generally, we hope that this study serves to identify the problem areas in mygalomorph classification and sets a course for a future research program delving into the systematics, taxonomy, and classification of this remarkable group of spiders.

### Summary of Nomenclatural Changes

The subfamily Euctenizinae is removed from the Cyrtaucheniidae and is elevated to the rank of family (NEW RANK); it includes the subfamilies and genera listed below. The subfamily Apomastinae (NEW SUBFAMILY) is established to accommodate *Myrmekiaphila, Aptostichus* and *Apomastus*. Nomenclatural changes are to be attributed to Bond and Hedin.

Euctenizidae Raven, 1985 (NEW RANK)

Apomastinae (NEW SUBFAMILY) Bond and Hedin

urn:lsid:zoobank.org:act:5C533E5D-0359-45F8-B37E-3BA34CC66303

*Apomastus* Bond and Opell, 2002

*Aptostichus* Simon, 1891

*Myrmekiaphila* Atkinson, 1886

Euctenizinae Raven, 1985

*Promyrmekiaphila* Schenkel, 1950

*Neoapachella* Bond and Opell, 2002

*Entychides* Simon, 1888

*Eucteniza* Ausserer, 1875

The following genus is transferred from the family Cyrtaucheniidae to the Nemesiidae:

*Kiama* Main & Mascord, 1969

## Supporting Information

Text S1 Morphological characters scored. Supporting data files in Nexus file format can be downloaded from the Dryad Data repository http://dx.doi.org/10.5061/dryad.7sq2j.
(DOCX)

Figure S1 **18S rRNA trees.** A. Bayesian; B. Likelihood.
(TIF)

Figure S2 **28S rRNA trees.** A. Bayesian; B. Likelihood.
(TIF)

Figure S3 **18S/28S rRNA trees.** A. Bayesian; B. Likelihood.
(TIF)

Figure S4 **EF1G trees.** A. Bayesian; B. Likelihood.
(TIF)

Figure S5 **Combined genes (18S/28S/EF1G).** A. Bayesian; B. Likelihood.
(TIF)

Figure S6 **Total Evidence (genes + morphology).** A. Bayesian; B. Likelihood.
(TIF)

Figure S7 **Morphological trees.** A. Bayesian; B. Likelihood.
(TIF)

Table S1 Exemplar taxa, location information, voucher numbers, and GenBank accession numbers for mygalomorph higher classification studies (this study, Bond and Hedin 2006, and Hedin and Bond 2006).
(DOC)
