# Supplementary material for: A Reconsideration of the Classification of the Spider Infraorder Mygalomorphae (Arachnida: Araneae) Based on Three Nuclear Genes and Morphology
Source: PLoS One. 2012 Jun 19;7(6):e38753. doi: 10.1371/journal.pone.0038753 (PMC3378619; doi:10.1371/journal.pone.0038753)
Supplement: Text S1 — Morphological characters scored. Supporting data files in Nexus file format can be downloaded from the Dryad Data repository http://dx.doi.org/10.5061/dryad.7sq2j. (DOCX) [file pone.0038753.s001.docx]

Characters and character states from (Bond and Opell, 2002; Bond and Hedin 2006).

1. Thorax: flat (0); sloping (1).

2. Caput: low (0); high (1).

3. Eye tubercle: absent (0); present, low (1); present, high (2).

4. Fovea: narrow (0); intermediate width and shallow (1); wide and deep (2).

5. Fovea: longitudinal (0); recurved (1); procurved (2); transverse (3).

6. Eyes: AME and PME subequal in diameter (0); AME diameter much larger than PME (1); PME much greater in diameter than AME.

7. Abdomen: without mottled striping (0); with mottled striping (1).

8. Ocular area: normal (1); occupies at least 2/3’s of the cephalic region of carapace (1).

9. Female carapace: not hirsute (0); hirsute (1).

10. Sternum shape: widest at coxae III and narrowing anteriorly; sides roughly parallel (1); rounded (2).

11. Sternum: wide, almost round (0); long and slender (1); normal (2).

12. Posterior sternal sigilla: positioned in lateral margins (0); positioned medially (1).

13. Posterior sternal sigilla: small and concentric (0); large and concentric (1); large with anterior margin distorted.

14. Labium: subquadrate (0); wider than long (1); longer than wide (2).

15. Labium: setae normal, not modified as cuspules (0); a few setae modified as cuspules (1); many setae modified as cuspules.

16. Palpal endite cuspules: absent (0); large patch restricted to proximal inner margin (1); distributed uniformly across face of endite.

17. Serrula: absent (0); present (1).

18. Rastellum: absent (0); consisting of large spines, not on a mound (1); spines on a distinct process (2).

19. Posterior edge of male carapace: aspinose (0); with a distinct fringe of heavy spines (1).

20. Posterior margin of cephalothorax: sclerotization normal (0); sclerotization light (1).

21. Fangs: long and slender (0); short and thick (1).

22. Anterior legs: subequal to posterior legs in length and circumference (0); shorter and more slender than posterior legs (1).

23. Tarsi: normal (0); stout, swollen (1).

24. Palpal endites: longer than wide (0); subquadrate (1).

25. Male tarsus IV: straight (0); slightly curved (1).

26. Male tarsus I: integral (0); pseudosegmented (1).

27. Inferior tarsal claw (ITS): present, normal in size (0); reduced in size (1); absent (2).

28. ITS: edentate (0); dentate (1).

29. Tarsus: normal length (0); very short (1).

30. Superior tarsal claw (STC) IV dentition: few teeth (0); many teeth, more than four (1).

31. STC I and palp: males and females with a single row of teeth, prolateral displacement of female palpal tooth row minimal (0); male and females with a

single row of teeth, evident prolateral displacement of palpal row distally, basal teeth on medial keel (1); male and female with one strong basal tooth,

sometimes with a few minute teeth (2); male and female with two rows of teeth (3); male and female claws edentate (4).

32. STC I basal tooth: normal, unmodified (0); elongate and bifid (1).

33. Scopulae: absent (0); light (1); dense (2).

34. Scopulae: absent (0); present, symmetrical (1); present, asymmetrical (2).

35. Male scopulae: present on leg IV (0); absent on leg IV (1).

36. Tarsal trichobothria: single zigzag row (0); wide band (1); reduced (2); single narrow row.

37. Tarsal organ: low, usually with concentric ridges (0); elevated (1).

38. Chelicerae: single tooth row with denticles (0); two rows of equally large teeth, lacking denticles (1).

39. Small cuticular projections on legs and spinnerets: absent (0); present (1).

40. Posterior lateral spinnerets (PLS) apical article: digitiform, long (0); digitiform, short (1); domed (2).

41. Posterior median spinnerets (PMS) spigot sizes: one size (0); two or more spigot sizes (1).

42. PMS spigot density: less than on PLS (0); subequal to PLS (1).

43. PMS: slender (0); stout (1).

44. Spigot shaft sculpturation: overlapping scale-like folds (0); upturned spines (1); smooth (2).

45. Apical article of PLS: one common spigot size (0); common spigot size with linear arrangement of 2–3 very stout spigots on apical-most aspect of the

distal article (1).

46. Pumpkiniform spigots: absent (0); present (1).

47. Fused spigots: absent (0); present (1).

48. Spigot bases: with invaginations (0); without (1).

49. Posterior leg spines: both dorsal and ventral (0); mostly dorsal (1).

50. Prolateral spine patch on female patella III: absent (0); large patch, more than three spines (1); small patch, 2–3 spines (2).

51. Prolateral spine patch on female patella IV: absent (0); present (1).

52. Preening combs on metatarsus IV: absent (0); present (1).

53. Spines on male cymbium: absent (0); present (1).

(continued on next page)

J.E. Bond, M. Hedin / Molecular Phylogenetics and Evolution 41 (2006) 70–85 83

Appendix B (continued)

54. Patch of long, dense spines on dorsal distal-most aspect of femur IV: absent (0); present (1).

55. Sparse patch of short stout spines on dorsal distal-most aspect of femur IV: absent (0); present (1).

56. Distal ventral spine patch on tarsus IV: absent (0); present (1).

57. Digging spines on anterior walking legs and pedipalps: absent (0); present (1).

58. Male mating clasper: without proximal, ventral excavation (0); with proximal, ventral excavation (1).

59. Male mating clasper tibia I: without distinct patch of short prolateral, distal spines (0); with a distinct patch of short prolateral, distal spines (1).

60. Male mating clasper tibia I: without mid-ventral megaspine (0); with a mid-ventral megaspine (1).

61. Male tibia II: without mid-ventral megaspine (0); with a mid-ventral megaspine (1).

62. Palpal bulb: normal (0); unique conformation (1).

63. Male palpal tibia: long and slender (0); short and stout (1).

64. Male palpal tibia: without a prolateral spine patch (0); with a prolateral spine patch (1).

65. Palpal femur: dorsal spine row absent (0); dorsal spine row present (1).

66. Embous: with serrations (0); without serrations (1). 67. Male palpal bulb: distal sclerite closed (0); distal sclerite open (1).

68. Excavation of prolateral palpal tibia with short thorn-like spines: absent (0); present (1).

69. Spermathecae: multilobular (0); single lobe (1); single lobe with laterally extended base (2).

70. Lateral base of spermathecae: not enlarged or absent (0); enlarged (1).

71. Burrow entrance: collar (0); thickened (‘‘cork’’) trapdoor (1); thin trapdoor (2); open burrow or exposed tube (3); funnel web (4).
