# Supplementary figures and images for: A Reconsideration of the Classification of the Spider Infraorder Mygalomorphae (Arachnida: Araneae) Based on Three Nuclear Genes and Morphology
Source: PLoS One. 2012 Jun 19;7(6):e38753. doi: 10.1371/journal.pone.0038753 (PMC3378619; doi:10.1371/journal.pone.0038753)

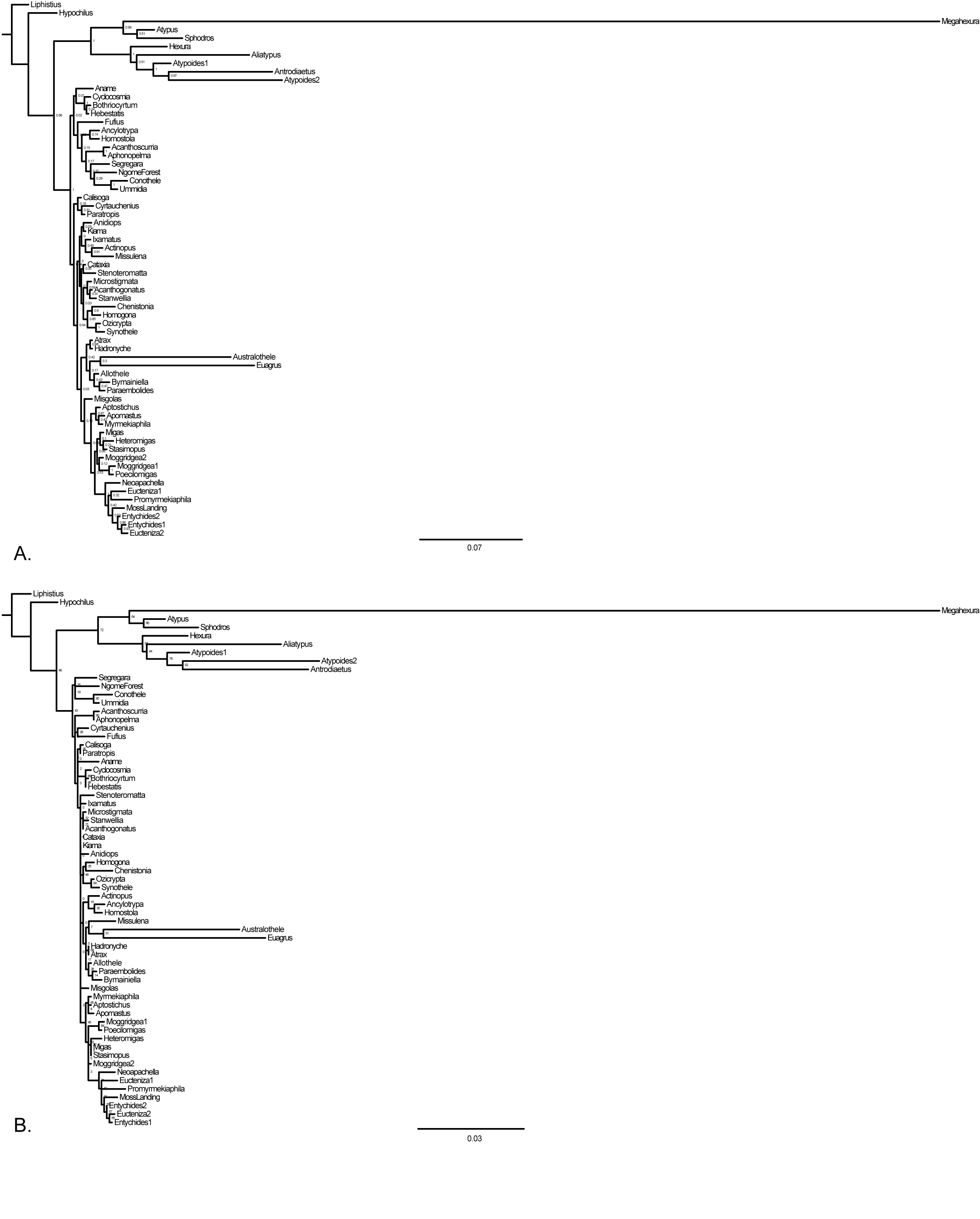

Supplement: Figure S1 — 18S rRNA trees. A. Bayesian; B. Likelihood. (TIF) [file pone.0038753.s002.tif]

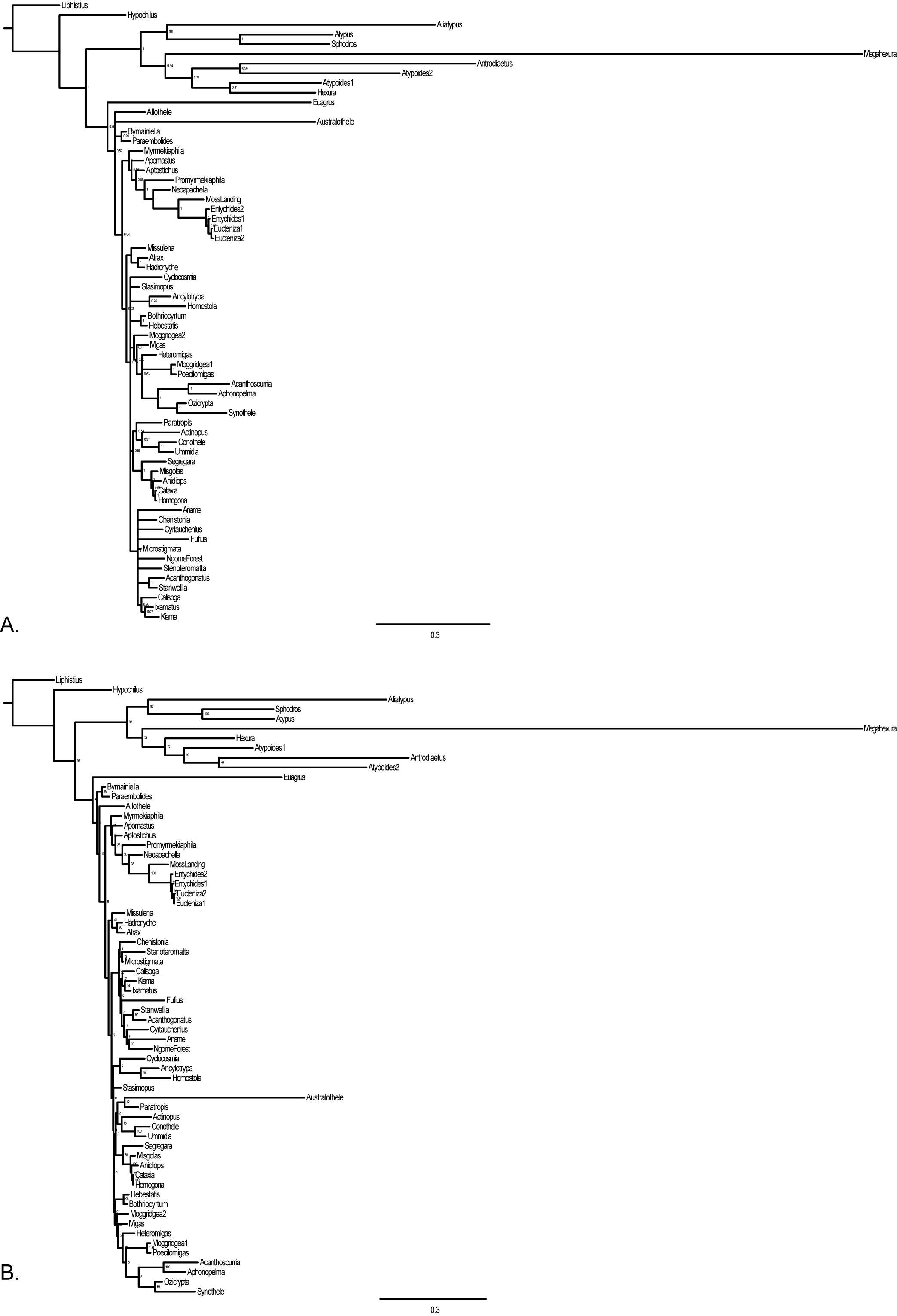

Supplement: Figure S2 — 28S rRNA trees. A. Bayesian; B. Likelihood. (TIF) [file pone.0038753.s003.tif]

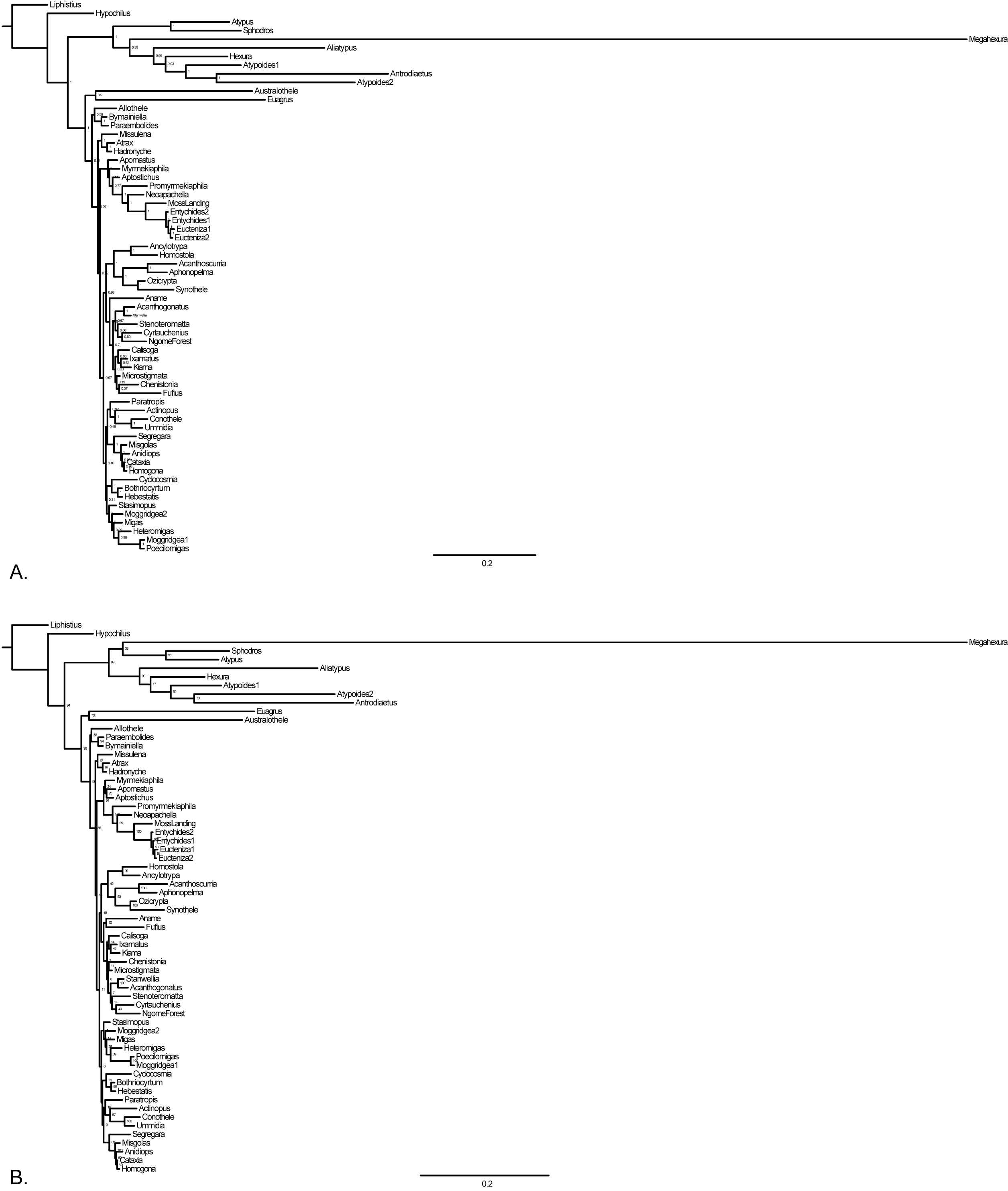

Supplement: Figure S3 — 18S/28S rRNA trees. A. Bayesian; B. Likelihood. (TIF) [file pone.0038753.s004.tif]

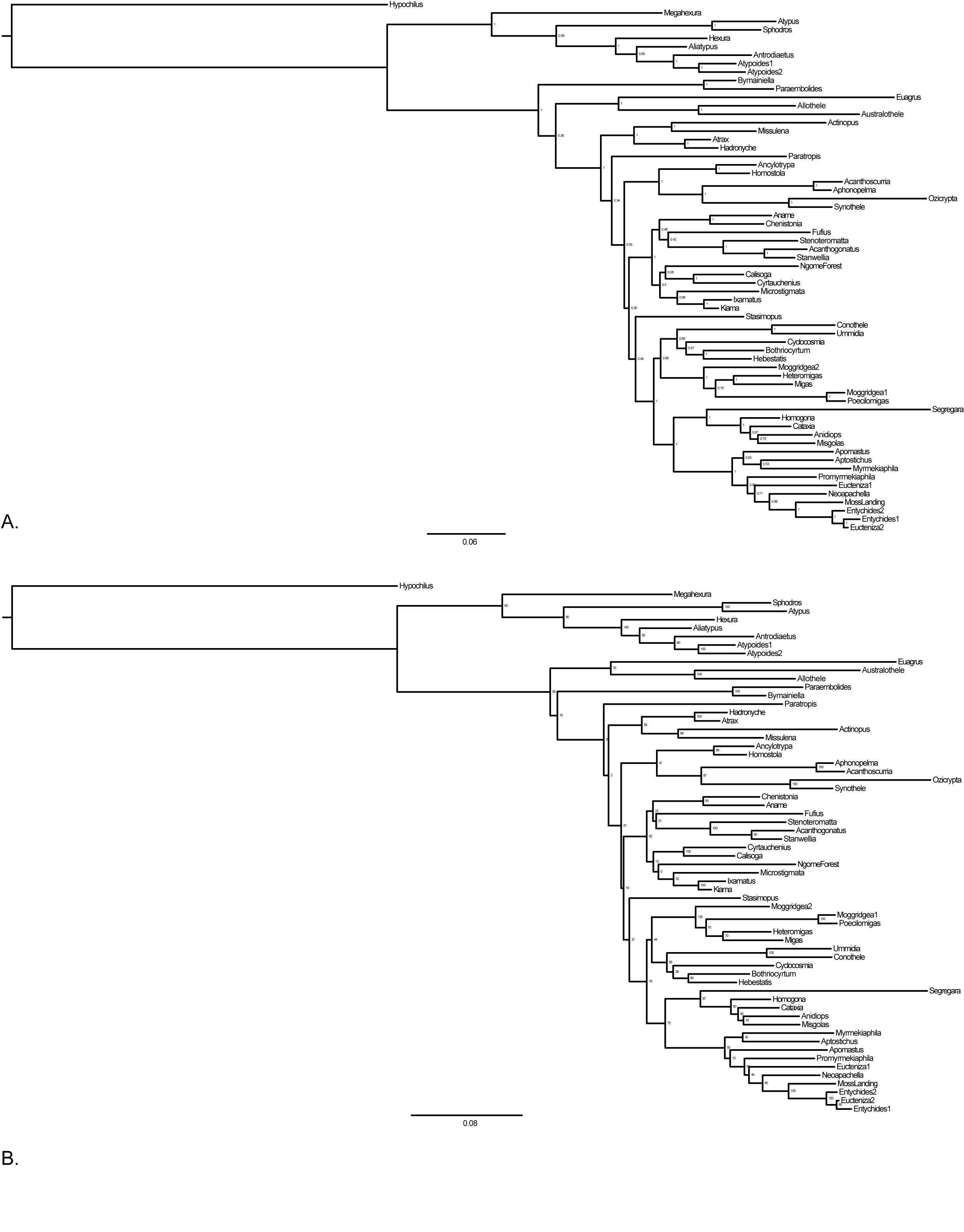

Supplement: Figure S4 — EF1G trees. A. Bayesian; B. Likelihood. (TIF) [file pone.0038753.s005.tif]

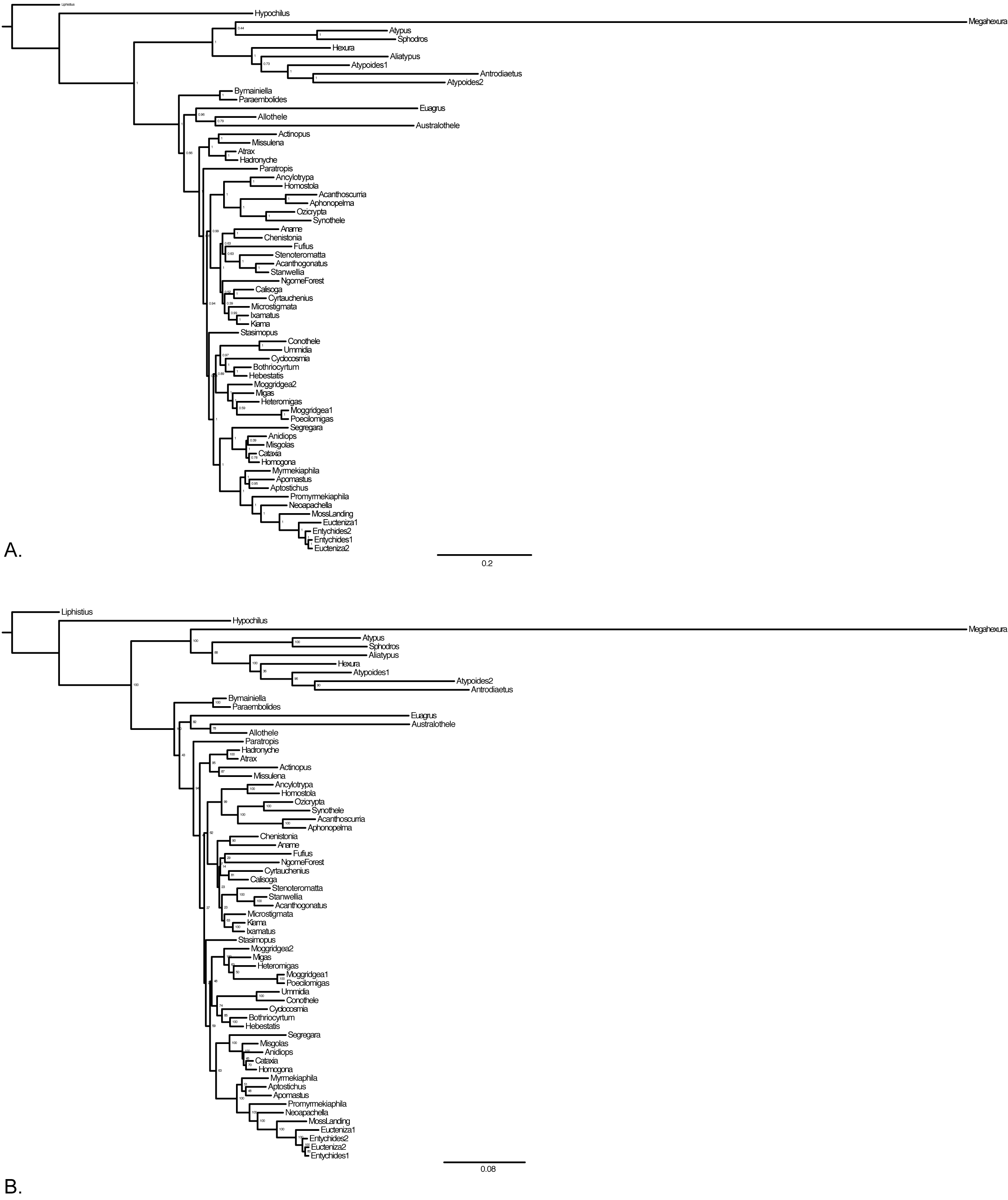

Supplement: Figure S5 — Combined genes (18S/28S/EF1G). A. Bayesian; B. Likelihood. (TIF) [file pone.0038753.s006.tif]

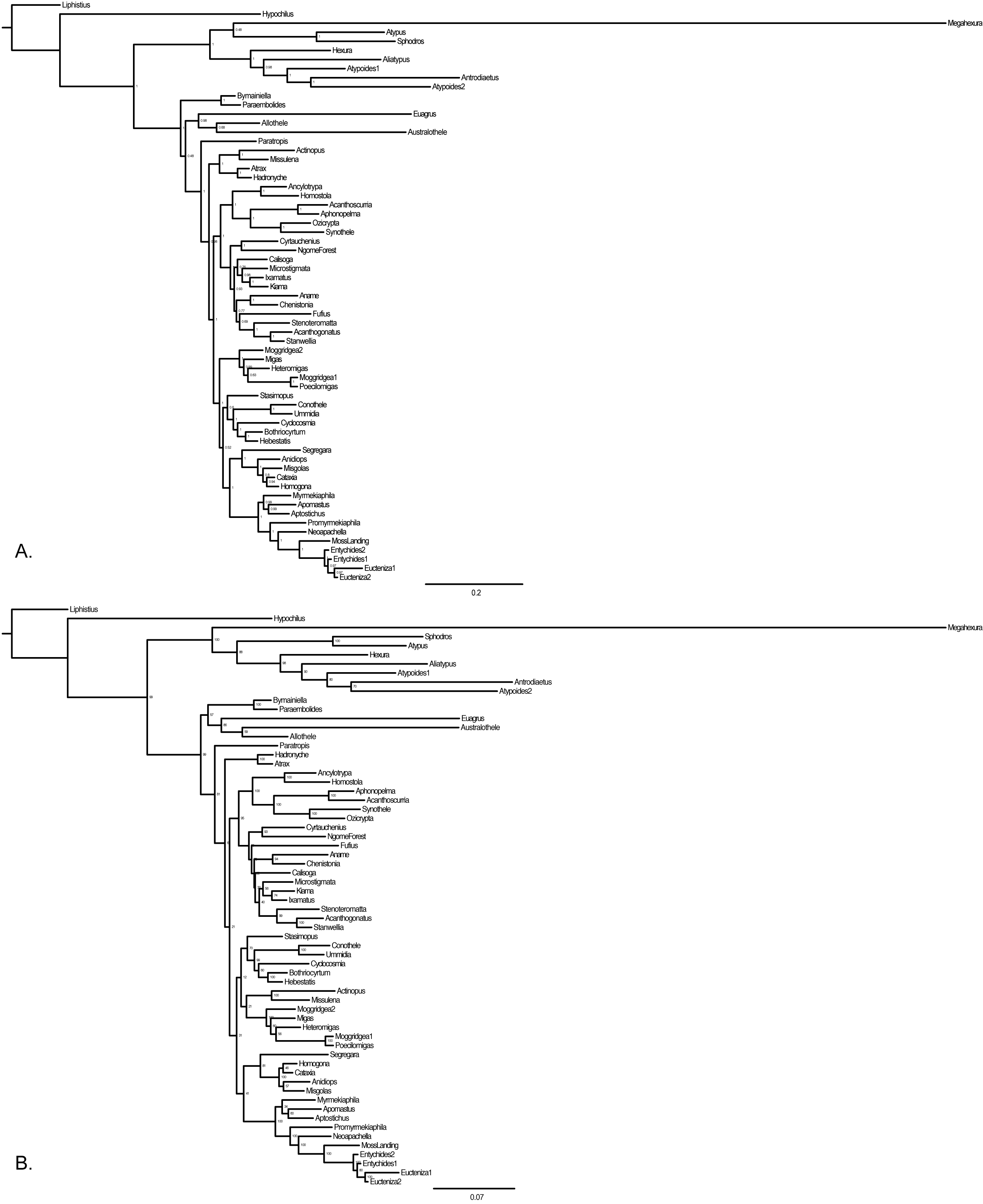

Supplement: Figure S6 — Total Evidence (genes + morphology). A. Bayesian; B. Likelihood. (TIF) [file pone.0038753.s007.tif]

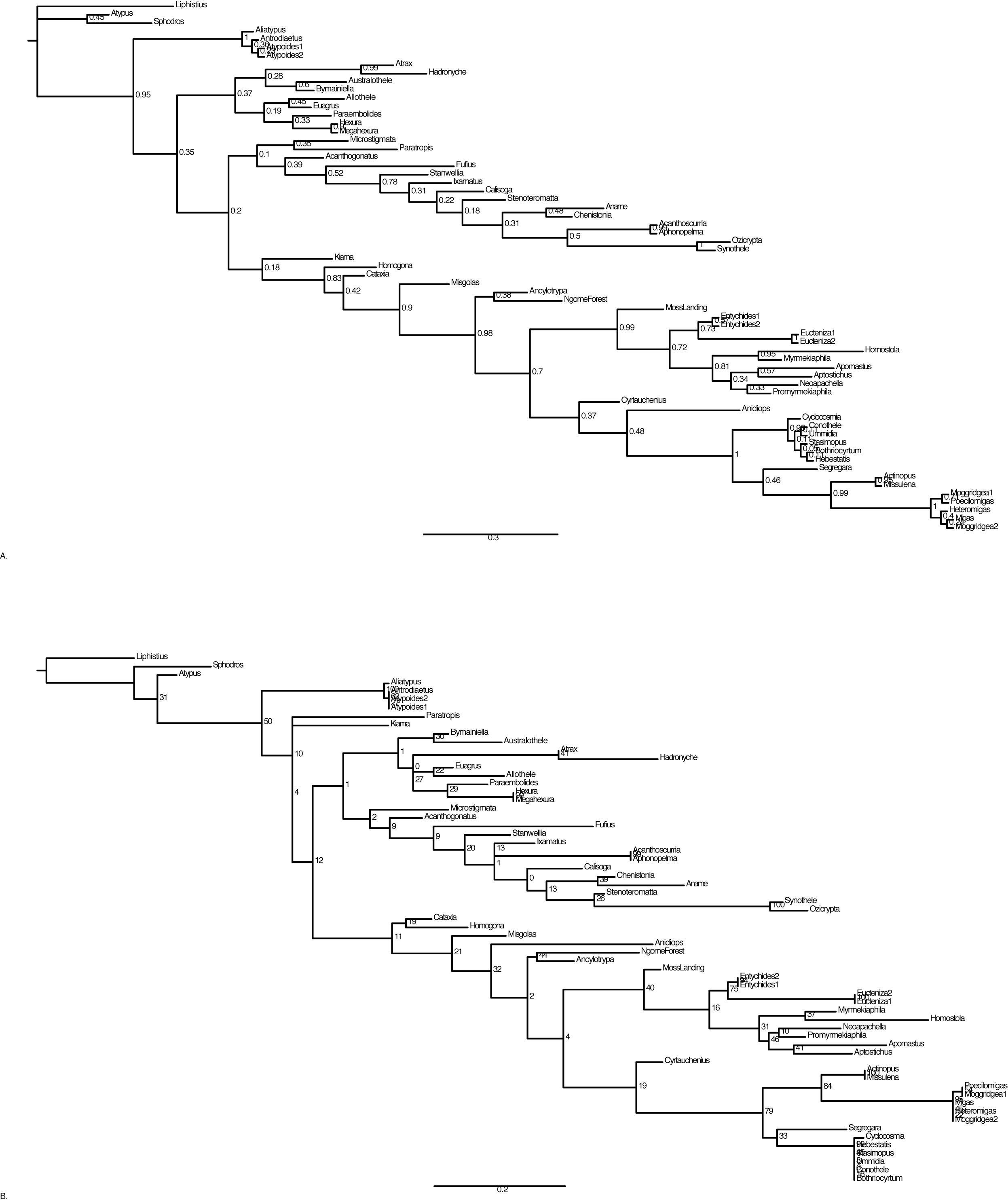

Supplement: Figure S7 — Morphological trees. A. Bayesian; B. Likelihood. (TIF) [file pone.0038753.s008.tif]
