# Supplementary material for: A Reconsideration of the Classification of the Spider Infraorder Mygalomorphae (Arachnida: Araneae) Based on Three Nuclear Genes and Morphology
Source: PLoS One. 2012 Jun 19;7(6):e38753. doi: 10.1371/journal.pone.0038753 (PMC3378619; doi:10.1371/journal.pone.0038753)
Supplement: Table S1 — Exemplar taxa, location information, voucher numbers, and GenBank accession numbers for mygalomorph higher classification studies (this study, Bond and Hedin 2006, and Hedin and Bond 2006). (DOC) [file pone.0038753.s009.doc]

**Table S1.** Supplementary Data Table. Exemplar taxa, location information, voucher numbers, and Genbank accession numbers. Complete list of mygalomorph taxa sequenced to data (includes samples from this paper, Hedin and Bond 2006 and Bond and Hedin 2006)

| **Family** | **Taxon/Author** | **Location** | **Voucher No.** | **28S Genbank No.** | **18S Genbank No.** | **EF1G Genbank No.** |
| --- | --- | --- | --- | --- | --- | --- |
|  |  |  |  |  |  |  |
| Liphistiidae  (Mesothelae) | *Liphistius malayanus* Abraham 1923 | Malayasia, Selangor | ATOL_GB | ARASP000057 (ATOL) |  |  |
| Liphistiidae  (Mesothelae) | *Liphistius bicoloripes*  Ono 1988 | Not available | ATOL_GB |  | AF007104 |  |
| Liphistiidae  (Mesothelae) | *Liphistius sp.* | Malayasia, Gua Tempurong Cave | MY1028 | MY1028-DQ639851 | MY1028-DQ639767 | ** |
|  |  |  |  |  |  |  |
| Hypochilidae (Araneomorphae) | *Hypochilus* sp. | USA |  | HEDIN-AF303505 | HAYASHI-AF062951 | AYOUB-DQ680342 |
|  |  |  |  |  |  |  |
| Atypidae | *Sphodros atlanticus*  Gertsch & Platnick 1980 | USA, South Carolina, vic. Clemson N34.7560 W82.8563 | MY0643 | MY0643 - DQ639852 |  | MY0643-  JQ358758 |
| Atypidae | *Sphodros abboti*  Walckenaer 1835 | USA, Florida, Gainesville N29.6462 W82.3581 | MY0026 |  | MY0026 - DQ639768 |  |
| Atypidae | *Atypus snetsingeri*  Sarno 1973 | USA, Pennsylvania | MY2282_2283 | MY2282 - DQ639853 | MY2283 - DQ639769 | MY2283-DQ680323 |
|  |  |  |  |  |  |  |
| Antrodiaetidae | *Aliatypus* sp. |  | MY0260_0482 | MY0260-DQ981738 | MY0482-  JX069738 | AYOUB-DQ680306 |
| Antrodiaetidae | *Antrodiaetus unicolor*  (Hentz 1842) | USA, Alabama, DeSoto St. Park N34.49877 W85.61774 | MY2015 | MY2015-DQ639854 | MY2015-DQ981691 | MY2015-  JQ358725 |
| Antrodiaetidae | *Antrodiaetus apachecus* Coyle 1971 | USA, Arizona, S Greer  N33.9958 W109.4647 | MY0118 |  | MY0118 - DQ639770 |  |
| Antrodiaetidae | *Antrodiaetus riversi*  (O.P.-Cambridge 1883) | USA, California, Bayview CG N38.94600 W120.09808 | MY2876 | MY2876 - DQ639855 | MY2876-DQ981698 | MY2876-  JQ358730 |
| Antrodiaetidae | *Atypoides riversi*  O.P.-Cambridge 1883 | USA, California, Trinity River  N40.6743 W122.8272 | MY0081 |  | MY0081 - DQ639771 |  |
| Antrodiaetidae | *Antrodiaetus gertschi*  (Coyle 1968) | USA, Oregon, Ashland N42.16319 W122.70294 | MY2894 | MY2894-DQ639856 | MY2894-DQ981696 | MY2894-  JQ358729 |
| Antrodiaetidae | *Atypoides gertschi*  Coyle 1968 | USA, California, vic Old Station N40.7335 W121.4475 | MY0432 |  | MY0432 - DQ639772 |  |
|  |  |  |  |  |  |  |
| Mecicobothriidae | *Hexura picea* Simon 1884 | USA, Oregon, E of Tidewater N44.40767 W123.88597 | MY0311 | MY0311-DQ639857 | MY0311-DQ639773 | MY0311-  JQ358745 |
| Mecicobothriidae | *Megahexura fulva* (Chamberlin 1919) | USA, California, vic Mariposa  N37.5039 W119.9941 | MY0113 | 28S | DQ639774 |  |
| Mecicobothriidae | *Megahexura fulva* (Chamberlin 1919) | USA, California, Alum Rock  N37.3967 W121.7976 | MY0113_0152 | MY0152-DQ639858 | MY0113-DQ639774 | MY0152-DQ680324 |
|  |  |  |  |  |  |  |
| Theraphosidae | *Acanthoscurria* sp. | Argentina, Formosa, Parque Nacional Rio Pilcomayo | MY2874 | MY2874-DQ639859 | MY2874-DQ639775 | MY2874-  JQ358721 |
| Theraphosidae | *Aphonopelma* sp. | USA, New Mexico, nr. Las Cruces N32.43120 W106.54921 | MY2464 | MY2464 - DQ639860 |  |  |
| Theraphosidae | *Aphonopelma reversum* Chamberlin 1940 | USA, California, near San Diego N32.5854 W116.7582 | MY0063 |  | MY0063 - DQ639776 | MY0063-DQ680311 |
| Theraphosidae | *Aphonopelma hentzi*  (Girard 1852) | Not available | Genbank | AY210803 |  |  |
| Theraphosidae | *Aphonopelma sp* | Not available | Genbank |  | ECRRN18 |  |
|  |  |  |  |  |  |  |
| Barychelidae | *Synothele arrakis* Raven 1994 | AUS, Western Australia, 38km N of Menzies S29.44338 E121.26000 | MY0841_2135 | MY2135-DQ639861 | MY2135-DQ639777 | MY0841-DQ680330 |
| Barychelidae | *Pisenor notius*  Simon 1889 | RSA, Guateng, Roodeplaat Nature Preserve S25.65095 E28.34425 | MY0507 | DQ639862 | DQ639778 |  |
| Barychelidae | *Ozicrypta* sp. | AUS, Queensland, Mt. Woowoonga S25.4319 E152.1115 | MY0839 | MY0839-DQ639863 | MY0839-DQ639779 | MY0839-DQ680326 |
| Barychelidae | *Ozicrypta filmeri*  Raven & Churchhill 1994 | AUS, Queensland, Amama Park, S26.36168 E152.64028 | MY2146 | DQ639864 | DQ639780 |  |
|  |  |  |  |  |  |  |
| Paratropididae | *Paratropis* sp | Ecuador, Jatun Sacha | MY2981 | MY2981-DQ639865 | MY2981-  JX069743 | MY2981-  JQ358755 |
|  |  |  |  |  |  |  |
| Dipluridae | *Euagrus josephus*  Chamberlin 1924 | MEX, Baja California Sur, El Triunfo N23.7843 W110.1223 | MY0147 | MY0147-DQ639866 | MY0147-DQ639781 | AYOUB-DQ680305 |
| Dipluridae | *Cethegus* sp. | AUS, Western Australia, Yorkrakine S31.37050 E117.58556 | MY2072 | DQ639867 | DQ639782 |  |
| Dipluridae | *Australothele jamiesoni* Raven 1984 | AUS, Queensland, Amama Park, S26.36168 E152.64028 | MY2084 | MY2084-DQ639868 | MY2084-  JX069739 | MY2084-  JQ358731 |
| Dipluridae | *Namirea planipes*  Raven 1984 | AUS, Queensland, Mt. Coot-Tha Park S27.47137 E152.97083 | MY2043 | DQ639869 | DQ639783 |  |
| Dipluridae | *Allothele australis*  (Purcell 1903) | RSA, Eastern Cape Province S33.1275 E26.6729 | MY0162_0575 | MY0575-DQ639870 | MY0575-DQ639784 | MY0162-DQ680310 |
|  |  |  |  |  |  |  |
| Hexathelidae | *Paraembolides cannoni* (Raven 1978) | AUS, Queensland, Lamington National Park S28.19347 E153.18722 | MY2083 | MY2083-DQ639871 | MY2083-DQ639785 | MY2083-  JQ358754 |
| Hexathelidae | *Bymainiella terraereginae* (Raven 1976) | AUS, Queensland, Lamington National Park S28.19863 E153.18722 | MY2045 | MY2045-DQ639872 | MY2045-DQ639786 | MY0883-DQ680312 |
| Hexathelidae | *Atrax robustus*  O. P.-Cambridge 1877 | AUS, Queensland, Lamington National Park S28.19347 E153.18722 | MY2049 | MY2049-DQ639873 | MY2049-DQ639787 | MY2049-  JQ358728 |
| Hexathelidae | *Hadronyche* sp. | AUS, New South Wales, Scalloway S34.73565 E150.79083 | MY2075 | MY2075-DQ639874 | MY2075-DQ639788 | MY0885-DQ680315 |
| Hexathelidae | *Porrhothele antipodiana*  (Walckenaer 1837) | New Zealand, Banks Peninsula, Otepatotu Res. S43.74917 E173.01583 | MY0858 | DQ639875 | ---- |  |
| Hexathelidae | *Macrothele sp* | Myanmar, Magwe, Shwesettaw Wildlife Sanctuary | MY1024 | DQ639876 | DQ639789 |  |
|  |  |  |  |  |  |  |
| Cyrtaucheniidae | *Homostola pardalina*  (Hewitt 1913) | RSA, Mpumalanaga Province S26.1774 E31.2169 | MY0530 | MY0530-DQ639877 | MY0530-DQ639790 | MY0530-  JQ358746 |
| Cyrtaucheniidae | *Ancylotrypa* sp. 1 | RSA, Guateng Province  S25.7268 E28.2377 | MY0515 | DQ639878 | DQ639791 |  |
| Cyrtaucheniidae | *Ancylotrypa* sp. 2 | RSA, Guateng Province  S25.6525 E28.3495 | MY0502 | DQ639879 | DQ639792 |  |
| Cyrtaucheniidae | *Ancylotrypa* sp. |  | MY0500 | MY0500-DQ639878 | MY0500-DQ639791 | MY0500-  JQ358724 |
| Cyrtaucheniidae | *Spiroctenus* sp. | RSA, Western Cape, Hwy N12 S33.68735 E22.26945 | MY0605 | DQ639880 | DQ639793 |  |
| Cyrtaucheniidae | *Spiroctenus* sp. | RSA, Western Cape, Hwy N12 S33.68735 E22.26945 | MY0610 | DQ639881 | ---- |  |
| Cyrtaucheniidae | *Fufius* sp. | French Guiana, Kourou N05.7217 W52.6929 | MY3399 | MY3399-DQ639882 | MY3399-DQ639794 | MY3399-  JQ358742 |
| Cyrtaucheniidae | *Acontius* sp. | Cameroon, 5 km N of Bafut on N11 | MY3400 | DQ639883 | DQ639795 |  |
| Cyrtaucheniidae | *Kiama lachrymoides*  Main & Mascord 1969 | AUS, New South Wales  S34.6993 E150.8064 | MY2094 | MY2094-DQ639884 | MY2094-DQ639796 | MY2094-  JQ358748 |
| Cyrtaucheniidae | *Aptostichus* sp. 1 | USA, California, Anza-Borrego State Park  N32.7118 W116.1160 | MY0264 | DQ639885 | DQ639797 |  |
| Cyrtaucheniidae | *Aptostichus* sp. 2 | USA, California, Winchester  N33.6771 W117.1158 | MY2595 | MY2595-DQ639886 | MY2595- | MY2595-  JQ358727 |
| Cyrtaucheniidae | *Apomastus kristenae*  Bond 2004 | USA, California, W of Laguna Beach N33.5529 W117.7678 | MY0720_2584 | MY0720-DQ639887 | MY0720-DQ639798 | MY2584-  JQ358726 |
| Cyrtaucheniidae | *Myrmekiaphila* sp. 1 | USA, Alabama, Bankhead NF  N34.3096 W87.39743 | MY2034 | DQ639888 | DQ639799 |  |
| Cyrtaucheniidae | *Myrmekiaphila fluviatilis* (Hentz 1850) | USA, Virginia, Cascades Rec Area N37.3538 W80.5999 | MY2234 | MY2234-DQ639889 | MY2234-DQ639800 | MY2234-  JQ358753 |
| Cyrtaucheniidae | *Neoapachella rothi*  Bond & Opell 2002 | USA, Arizona, near Greer N33.9955 W109.4672 | MY0079_0252 | MY0252 - DQ639890 | MY0079 – DQ639801 | MY0079-DQ680319 |
| Cyrtaucheniidae | *Promyrmekiaphila* sp. | USA, California, Glenn Co.  N39.6155 W122.5133 | MY0736 | MY0736-DQ639891 | MY0736-DQ639802 | MY0736-  JQ358757 |
| Cyrtaucheniidae | *Entychides arizonicus* Gertsch & Wallace 1936 | USA, Arizona, near Portal  N31.9330 W109.2720 | MY2281 | MY2281-DQ639892 | MY2281-DQ639803 | MY2281-  JQ358737 |
| Cyrtaucheniidae | *Entychides* sp. |  | MY3548 | MY3548- | MY3548- | MY3548-  JQ358738 |
| Cyrtaucheniidae | *Eucteniza rex*  (Chamberlin 1940) | USA, Texas, Hwy 83, 1.8 miles N jnct. Hwy 35 N27.78889 W99.45583 | TX6 | TX6-DQ639893 | TX6-DQ639804 | TX6-  JQ358739 |
| Cyrtaucheniidae | *Eucteniza* n. sp. | MEX, Baja California Sur, near La Paz  N24.1003 W110.2692 | MY2698 | MY2698-DQ639894 | MY2698-DQ639805 | MY2698-  JQ358740 |
| Cyrtaucheniidae | *Cyrtauchenius* sp. | Spain | MY3619 | MY3619-  JX069744 | MY3619-  JX069740 | MY3619-  JQ358736 |
| Cyrtaucheniidae | NEW GENUS | Moss Landing | MY3071_3072 | MY3072-DQ672620 | MY3071-  JX069742 | MY3071-  JQ358741 |
|  |  |  |  |  |  |  |
| Migidae | *Migas variapalpus*  Raven 1984 | AUS, Queensland, Lamington National Park S28.19347 E153.18722 | MY2104 | MY2104-DQ639895 | MY2104-DQ639806 | MY2104-  JQ358749 |
| Migidae | *Heteromigas terraereginae* Raven 1984 | AUS, Queensland, Mt. Woowoonga S25.4319 E152.1115 | MY2138 | MY2138-DQ639896 | MY2138-DQ639807 | MY2138-  JQ358744 |
| Migidae | *Moggridgea tingle*  Main 1991 | AUS, Western Australia, Stirling Range NP S34.4134 E117.9570 | MY2147 | MY2147-DQ639897 | MY2147-DQ639808 | MY2147-  JQ358752 |
| Migidae | *Moggridgea crudeni*  Hewitt 1913 | RSA, Northern Cape, .5 km East of N7 S30.64556 E18.05889 | MY0623 | MY0623-DQ639898 | MY0623- | MY0623-  JQ358751 |
| Migidae | *Poecilomigas abrahami*  (O.P.-Cambridge 1889) | RSA, Western Cape Prov.  S 33.9703 E 23.5389 | MY0598 | MY0598-DQ639899 | MY0598-DQ639809 | MY0598-  JQ358756 |
| Migidae | *Thyropeous* *mirandus*  Pocock 1895 | Madagascar, Fianaranstoa Prov. Massif de Andringitra S21.96400 E46.933167 | MY2859 | DQ639900 | DQ639810 |  |
|  |  |  |  |  |  |  |
| Ctenizidae | *Stasimopus mandelai* Hendrixson & Bond 2004 | RSA, Eastern Cape, Great Fish River Reserve S33.12755 E26.67287 | MY0557 | MY0557 - DQ639901 |  |  |
| Ctenizidae | *Stasimopus mandelai* Hendrixson & Bond 2004 | RSA, Northern Province, Rust de Winter S25.2171 E28.4926 | MY0161 |  | MY0161 - DQ639811 | MY0161-DQ680321 |
| Ctenizidae | *Stasimopus* sp. | RSA, Eastern Cape, Great Fish River Reserve S33.12755 E26.67287 | MY0565 | DQ639902 | ---- |  |
| Ctenizidae | *Cyclocosmia truncata*  (Hentz 1841) | USA, Alabama, Sipsey WA  N34.3409 W87.4710 | MY0457 | DQ639903 | DQ639812 |  |
| Ctenizidae | *Cyclocosmia truncata*  (Hentz 1841) | USA, Alabama, Borden Creek Trail N34.30959 W87.39433 | MY2033 | DQ639904 | ---- |  |
| Ctenizidae | *Cyclocosmia loricata* |  | MY3547 |  |  | MY3547-  JQ358735 |
| Ctenizidae | *Hebestatis theveneti*  (Simon 1891) | USA, California, N Mariposa  N37.5039 W119.9940 | MY0278 | MY0278 – DQ639905 |  |  |
| Ctenizidae | *Hebestatis theveneti*  (Simon 1891) | USA, California, NE Mariposa N37.5032 W119.9673 | MY0078 |  | MY0078 - DQ639813 |  |
| Ctenizidae | *Hebestatis theveneti*  (Simon 1891) |  | MY2635 |  |  | MY2635-  JQ358743 |
| Ctenizidae | Bothriocyrtum californicum (O.P.-Cambridge 1874) | USA, California, San Diego  N 32.4672 W117.0436 | MY0066 | MY0066-DQ639906 | MY0066-DQ639814 | AYOUB-DQ680304 |
| Ctenizidae | *Ummidia sp* | USA, North Carolina, Midland  N35.21800 W80.57670 | MY2042 | MY2042-DQ639907 |  |  |
| Ctenizidae | *Ummidia sp* | USA, Arizona, Ponderosa Pk  N34.4561 W112.4896 | MY0149 |  | MY0149 - DQ639815 | MY0149-DQ680322 |
| Ctenizidae | *Ummidia* sp. | USA, North Carolina, N of Newbern N35.1825 W77.0754 | MY2313 | DQ639908 | ---- |  |
| Ctenizidae | *Conothele* sp. | AUS, Western Australia, SE of Nungarin S31.29432 E118.19944 | MY2070 | MY2070-DQ639909 | MY2070-DQ639816 | MY2070-  JQ358734 |
|  |  |  |  |  |  |  |
| Actinopodidae | *Actinopus* sp. | Argentina, Buenos Aires, Parque Leloir | MY2873 | DQ639910 | DQ639817 | MY2873  JQ358722 |
| Actinopodidae | *Missulena* sp. |  | MY0861_2086 | MY2086- | MY2086- | MY0861-DQ680325 |
|  |  |  |  |  |  |  |
| Idiopidae | *Cataxia* sp. | AUS, Queensland, Mt. Coot-Tha Park S27.47137 E152.97083 | MY2044 | MY2044-DQ639911 | MY2044-DQ639818 | MY2044-  JQ358732 |
| Idiopidae | *Arbanitis* sp. | AUS, Western Australia, Stirling Range National Park S34.3677 E118.24222 | MY2053 | DQ639912 | DQ639819 |  |
| Idiopidae | *Misgolas hubbardi*  Wishart 1992 | AUS, New South Wales, Scalloway S34.73565 E150.79083 | MY2093 | MY2093-DQ639913 | MY2093-DQ639820 | MY2093-  JQ358750 |
| Idiopidae | *Misgolas* sp. | AUS, New South Wales, Minnanurra Rainforest S34.63455 E150.72762 | MY2089 | DQ639914 | ---- |  |
| Idiopidae | *Neohomogona (= Cataxia) stirlingi* (Main, 1985) | AUS, Western Australia, Stirling Range NP S34.3677 E118.2421 | MY2148 | DQ639915 | DQ639821 |  |
| Idiopidae | *Homogona* (= *Cataxia)* *pulleinei* (Rainbow 1914) | AUS, Queensland, Lamington National Park S28.19347 E153.18722 | MY0897_2081 | MY2081 - DQ639916 | MY0897-DQ639822 | MY0897-DQ680316 |
| Idiopidae | *Homogona* (= *Cataxia)* *pulleinei* (Rainbow 1914) | AUS, Queensland, Lamington National Park S28.1986 E153.1872 | MY0897_2081 |  | MY897 - DQ639822 |  |
| Idiopidae | *Anidiops manstridgei*  Pocock 1897 | AUS, Western Australia, Korrelocking S31.20172 E117.47722 | MY2152 | MY2152-DQ639917 | MY2152-DQ639823 | MY0945-DQ680313 |
| Idiopidae | *Aganippe cupulifex*  Main 1957 | AUS, Western Australia, Wungong Damn S32.19487 E116.05917 | MY2056 | DQ639918 | DQ639824 |  |
| Idiopidae | *Eucyrtops eremaea*  Main 1957 | AUS, Western Australia, E of Southern Cross S31.2887 E119.6858 | MY2071 | DQ639919 | DQ639825 |  |
| Idiopidae | *Idiops* sp. | RSA, Northern Province  S 24.7895 E 28.4042 | MY0189 | DQ639920 | DQ639826 |  |
| Idiopidae | *Ctenolophus* sp. | RSA, Mpumalanga, Songimvelo S26.0400 E31.0026 | MY0321 | DQ639921 | DQ639827 |  |
| Idiopidae | *Segregara* sp. | RSA, Western Cape Province  S 32.3347 E 22.4747 | MY0192_0604 | MY0604-DQ639922 | MY0604-DQ639828 | MY0192-DQ680329 |
| Idiopidae | *Segregara paucispinulosus* (Hewitt 1915) | RSA, Kwa-Zulu Natal, Vryheid S27.75033 E30.76847 | MY0539 | DQ639923 | ---- |  |
| Idiopidae | *Gorgyrella* sp | RSA, West Cape Province, E Citrusdal S32.6030 E19.0402 | MY0183 | DQ639924 | DQ639829 |  |
|  |  |  |  |  |  |  |
| Nemesiidae | *Calisoga (= Brachythele) theveneti* Simon 1981 | USA, California, vic Mariposa  N37.5039 W119.9941 | MY0077 | MY0077-DQ639925 | MY0077-DQ639830 | MY0077-DQ680314 |
| Nemesiidae | *Ixamatus* sp. juv. | AUS, Queensland, near Mudgeerba S28.13462 E153.28611 | MY2102 | MY2102-DQ639926 | MY2102-DQ639831 | MY2102-  JQ358747 |
| Nemesiidae | *Aname* sp. | AUS, Western Australia, N of Leonora S28.10172 E125.90417 | MY2121 | MY2121-DQ639927 | MY2121-DQ639832 | MY2121-  JQ358723 |
| Nemesiidae | *Aname* sp. | AUS, Western Australia, SE of Nungarin S31.29432 E118.19944 | MY2065 | DQ639928 | DQ639833 |  |
| Nemesiidae | *Kwonkon goongarriensis*  Main 1983 | AUS, Western Australia, 38km N of Menzies S29.44338 E121.26000 | MY2061 | DQ639929 | DQ639834 |  |
| Nemesiidae | *Teyl luculentus* Main 1975 | AUS, Western Australia, Avon Valley NP S31.59755 E116.24694 | MY2063 | DQ639930 | DQ639835 |  |
| Nemesiidae | *Pseudoteyl vancouveri*  Main 1985 | AUS, Western Australia, West Cape Howe NP S35.0950 E117.6267 | MY2060 | DQ639931 | DQ639836 |  |
| Nemesiidae | *Chenistonia (=Aname) tepperi* (Hogg 1902) | AUS, Western Australia, Wungong Damn S32.19487 E116.05917 | MY2096 | MY2096-DQ639932 | MY2096-DQ639837 | MY2096-  JQ358733 |
| Nemesiidae | *Namea flavomaculata* (Rainbow & Pulleine 1918) | AUS, Queensland, Lamington National Park S28.19347 E153.18722 | MY2046 | DQ639933 | DQ639838 |  |
| Nemesiidae | *Entypesa schoutedeni*  Benoit 1965 | RSA, Mpumalanga, Barberton S25.78627 E31.06075 | MY0528 | DQ639934 | DQ639839 |  |
| Nemesiidae | *Iberesia machadoi*  Decae & Cardosa 2005 | Portugal, Ribeira de Limas | MY1008 | DQ639935 | DQ639840 |  |
| Nemesiidae | *Entypesa* sp. | Madagascar, Toamasina, Parc National Andasibe S18.94394 E48.417556 | CAS580 | DQ639936 | DQ639841 |  |
| Nemesiidae | *Stanwellia hoggi*  (Rainbow 1914) | AUS, New South Wales, N of Stanwell Park S34.22273 E150.99000 | MY0912_2092 | MY2092-DQ639937 | MY2092-DQ639842 | MY0912-DQ680320 |
| Nemesiidae | *Acanthogonatus campanae* Legendre and Calderon 1984 | CHILE, Region VIII, Parque Nacional Nahuelbuta S37.825167 W73.03533 | CAS539 | CAS539-DQ639938 | CAS539-DQ639843 | CAS539-  JQ358720 |
| Nemesiidae | *Acanthogonatus nahuelbuta* Goloboff 1995 | CHILE, Region VIII, Parque Nacional Nahuelbuta S37.80333 W73.024722 | CAS542.1 | DQ639939 | DQ639844 |  |
| Nemesiidae | *Stenoteromatta palmer* Goloboff 1995 | Argentina, Entre Rios, Parque Nacional El Palmer | MY2872 | MY2872-DQ639940 | MY2872-DQ639845 | MY2872-  JQ358759 |
| Nemesiidae | *Stenoteromatta palmer* Goloboff 1995 | Argentina, Entre Rios, Parque Nacional El Palmer | MY2875 | DQ639941 | ---- |  |
| Nemesiidae | gen. nov. | MEX, San Luis Potosi, Municipio Ciudad Valles N22.1854 W98.9869 | MY3398 | DQ639942 | DQ639846 |  |
| Nemesiidae | *Hermacha* sp. | RSA, Northern Province, Nylsvlei Nature Reserve S24.64922 E28.67360 | MY0512 | DQ639943 | DQ639847 |  |
| Nemesiidae | gen. nov. | RSA, Eastern Cape Prov., Moss Landing  S33.1275 E 26.6729 | MY0536 | DQ639944 | DQ639848 |  |
| Nemesiidae | gen. nov. | RSA, Kwa-Zulu Natal Prov., Ngome Forest  S27.8200 E31.4175 | MY0175_0551 | MY0551-DQ639945 | MY0551-DQ639849 | MY0175-DQ680309 |
|  |  |  |  |  |  |  |
| Microstigmatidae | *Microstigmata longipes* (Lawrence 1938) | RSA, Kwa-Zulu Natal Prov., Ngome Forest  S27.8200 E 31.4175 | MY0165_0543 | MY0543 – DQ639946 | MY0165 – DQ639850 | MY0165-DQ680317 |

**Notes:** The 18S sequences of MY 536 and MY 551 (RSA, new genus) are identical, but included in combined analysis for sake of matrix completeness (i.e., the 28S sequences from these specimens are different). The same is true for MY 502 and MY 515 (*Ancylotrypa*).

For 28S, duplicate (identical) sequences are as follows: MY551 = MY546, 547 (RSA, new genus); MY1008=MY1009 (*Iberesia machadoi)*; MY77 = MY281 (*Calisoga)*; MY2044=MY2105 (*Cataxia)*; MY2042=MY2041 (*Ummidia*); MY515=MY500 (*Ancylotrypa)*; MY2075=MY2091 (*Hadronyche*); MY2043=MY2102 (*Nameria*). These duplicate sequences were not included in any phylogenetic analyses. For 18S, duplicate (identical) sequences are as follows: MY551=MY546, 547, 317 (RSA, new genus); MY1008=MY1009 (*Iberesia machadoi*); MY77=MY281 (*Calisoga*); MY2044=MY2105 (*Cataxia*); MY66=MY111 (*Bothriocyrtum californicum*); MY736=MY2269, MY2271 (*Promyrmekiaphila);* MY720=MY228 *(Apomastus*); MY515=MY500 (*Ancylotrypa*); MY530=MY314 (*Homostola*); MY575=MY162 (*Allothele australis*); MY147=MY92 (*Euagrus*); MY113=MY73 (*Megahexura fulva*); MY311=MY76, 88 (*Hexura picea*). These duplicate sequences were not included in any phylogenetic analyses. The taxonomic identify and location information for all duplicate sequences is available upon request from the authors.
